# HP1β and H3K9me3 Regulate Olfactory Receptor Choice and Transcriptional Identity

**DOI:** 10.3390/ijms27072958

**Published:** 2026-03-24

**Authors:** Martín Escamilla-del-Arenal, Rachel Duffié, Hani Shayya, Valentina Loconte, Axel Ekman, Lena Street, Kevin Monahan, Carolyn Larabell, Marko Jovanovic, Stavros Lomvardas

**Affiliations:** 1 Department of Biochemistry and Molecular Biophysics, Mortimer B. Zuckerman Mind Brain and Behavior Institute, Columbia University, New York, NY 10027, USA; 2 Department of Anatomy, School of Medicine, University of California San Francisco, San Francisco, CA 94158, USA; 3 Lawrence Berkeley National Laboratory, Molecular Biophysics and Integrated Bioimaging Division, Berkeley, CA 94720, USA; 4 B24 Beamline, Diamond Light Source, Harwell Science and Innovation Campus, Didcot OX11 0DE, UK; 5 Department of Biological Sciences, Fairchild Center, Columbia University, New York, NY 10027, USA; 6 Nelson Biological Laboratories, Department of Molecular Biology and Biochemistry, Rutgers, The State University of New Jersey, Piscataway, NJ 08854, USA

**Keywords:** olfaction, epigenetics, chromatin, HP1, transcriptional regulation, cell identity

## Abstract

Diverse epigenetic regulatory mechanisms ensure and modulate cellular diversity. The histone 3 lysine 9 me3 (H3K9me3) post-translational modification participates in silencing lineage-inappropriate genes by restricting access of transcription factors and other regulatory proteins to genes that control cell fate. Mouse olfactory sensory neurons (OSNs) select one olfactory receptor (OR) gene out of 2600 possibilities. This monoallelic and stochastic OR choice occurs as OSNs differentiate and undergo dramatic changes in nuclear architecture. OR genes from different chromosomes converge into specialized nuclear bodies and chromatin compartments, as H3K9me3 and chromatin binding proteins including heterochromatin protein 1 (HP1) are incorporated. In this work, we have uncovered an unexpected role for HP1β in OR choice and neuronal identity that cannot be rescued by HP1α in vivo. With the use of a conditional knock-in mouse model, that after CRE expression replaces HP1β with HP1α, we observe changes in H3K9me3 levels and DNA accessibility over OR gene clusters. These changes alter the expression patterns that partition the mouse olfactory epithelium into five OR expression zones, which results in a reduced OR repertoire that leads to a loss of olfactory sensory neuron diversity. We propose that HP1β modulates the competition of OR promoters for enhancers to promote receptor diversity by establishing repression gradients in a zonal fashion.

## 1. Introduction

Distinct mechanisms have evolved to generate cell diversity in the animal kingdom. In mammals, two remarkable examples where diversity is achieved by different processes [1] are VDJ recombination in the immune system [reviewed in [2]] and OR choice in the olfactory system. Monoallelic and stochastic gene choice are essential for dosage compensation, cell differentiation and cell identity [3,4,5,6,7,8,9,10]. Stochastic choice is particularly important for neuronal diversity [5,7,8,9]. Decoding the mechanisms that govern stochastic choice will not only clarify this fascinating mode of gene expression regulation but also reveal fundamental principles of eukaryotic gene regulation.

Singular OR choice is essential for proper function of the olfactory system, as it determines the odor(s) from the environment to which the OSN will respond and ensures axonal wiring to a stereotyped glomerulus in the olfactory bulb [11]. While in a given OSN, OR choice is random and stochastic, and each OR is restricted to one of five anatomical regions in the Main Olfactory Epithelium (MOE). This restricted expression to a defined physical domain is the first level at which OR choice is regulated. Nuclear organization and heterochromatinization of the OR gene loci are essential for both silencing and enabling the activation of an OR allele [12,13,14]. OR genes can form interchromosomal contacts with at least 64 OR enhancers located throughout the genome [15,16]. OR gene choice is mediated by an enhancer hub that facilitates the transcription of the selected OR [16,17]. Transcriptional upregulation of one OR allele serves to silence other OR alleles [17]. Seminal work shows that heterochromatin regulates OR choice [18]. The transcriptionally silent OR genes are marked by H3K9me2 and acquire H3K9me3 upon differentiation [19]. The first insight that H3K9me3-based heterochromatin OR gene silencing was necessary to generate gene diversity came from the G9a and GLP histone methyltransferases (HMT) conditional KO alleles [18]. The absence of G9a and GLP, necessary for H3K9me2 incorporation, interfered with normal monogenic receptor expression and led to a decrease in OSN diversity [18]. Recent work has revealed zonal differences in H3K9me3 deposition patterns in the MOE [14], raising the possibility that heterochromatin plays a fundamental role in zonal OR choice and neuronal identity. However, the mechanism by which H3K9me3 impacts OR gene choice remains unknown.

In this work, we focus on the HP1 family of proteins, particularly HP1β, an H3K9me3 reader [20] that colocalizes with the OR nuclear compartment [12]. Mammalian cells contain three closely related HP1 homologs, namely HP1α (*Cbx5*), HP1β (*Cbx1*), and HP1γ (*Cbx3*) [21]. These HP1 paralogs share two highly conserved domains, an N-terminal chromo-domain and a C-terminal chromo shadow domain, separated by a hinge region (reviewed in [22]). HP1 proteins are evolutionarily conserved, nonhistone chromosomal proteins that bind H3K9me2/3 and regulate heterochromatin organization and epigenetic gene silencing [20,21,22,23]. While HP1α is found exclusively in silenced heterochromatic regions, some reports also implicate HP1β and HP1γ in gene activation (reviewed in [24,25]). *Cbx1*−/− mutants exhibit peri-natal lethality, which can neither be rescued by HP1α nor HP1γ [26]. HP1β but not HP1α is required for the proliferation of pluripotent embryonic stem cells in mice [27]. Some evidence assigns unique functions to each of the three paralogs, but whether these paralogs function cooperatively or separately in vivo is poorly understood. A generally accepted model for heterochromatin formation proposes that histone methyltransferases (HMT) facilitate HP1 incorporation into chromatin by methylating histone 3 at lysine 9 [28]. HP1 through H3K9me2/3 recognition and self-oligomerization [24] will promote interaction with other proteins, including HMT, ensuring compaction and spreading of the repressed chromatin state. Chromatin remodeling promotes or restricts access of DNA-interacting proteins to their target sequences, which in turn facilitates or prevents the expression of lineage specific genes [29,30]. In addition, recent studies in *Schizosaccharomyces pombe*, *Drosophila* and *Mus musculus* have reported the unexpected ability of HP1 proteins to aggregate into membrane-less nuclear bodies [31,32,33]. This liquid phase separation represents a new biophysical model for heterochromatin compartmentalization and gene repression. Together, these data support a model where HP1 proteins primarily act in spreading the repressive state of chromatin. However, HP1 protein function in establishing cell diversity and cell identity is not well-understood.

In the current study, we hypothesized that HP1β would play an essential role in setting up heterochromatin in OSNs during OR choice. We tested whether HP1α and HP1β have redundant or unique roles in OR heterochromatinization. We demonstrate that in the absence of HP1β, high levels of H3K9me3 fail to incorporate in neurons from the MOE, regulating OR choice and transcriptional identity. HP1α and HP1β proteins were restricted to different nuclear compartments, highlighting the compartmentalization of these two proteins and supporting their distinct function. To overcome peri-natal lethality upon deletion of the *Cbx1* gene, we designed a knock-in mouse model. After CRE expression, HP1α protein is expressed under the control of the endogenous *Cbx1* promoter, which rescues the lethality observed in the *Cbx1* KO mouse. In the absence of HP1β, however, we observe a decrease in H3K9me3 over OR clusters and a drop in genome-wide heterochromatin levels, impacting OR gene choice and transcriptional cell identity. We propose a model wherein HP1β regulates OR choice and OSN transcriptional identity.

## 2. Results

### 2.1. Developmental Kinetics of HP1α and HP1β Proteins During OSN Differentiation

**Figure 1:**
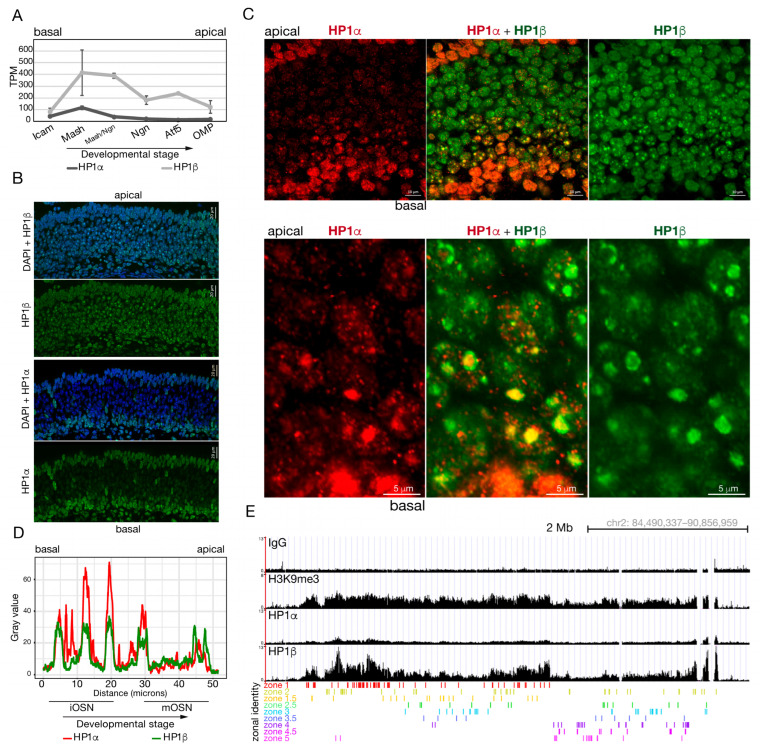
**HP1α and HP1β segregate into different compartments during OSN differentiation**. (**A**) RNA-seq of sorted cells at different points in olfactory sensory neuron differentiation. TPM (Transcript Per Million). Data from two biological replicates. Data from Duffié et al., 2025 [35]. (**B**) IF on Mouse Olfactory Epithelium (MOE) sections, using specific antibodies against HP1α and HP1β. Basal and apical localization of the tissue is indicated. Scale bar: 20 μm. (**C**) IF on MOE sections using specific antibodies against HP1α (red) and HP1β (green). Top images taken with a 40X lens shows HP1α and HP1β staining colocalize in immature Olfactory Neurons (iOSN). Scale bar: 10 μm. Bottom images taken with a 64X lens from a different part of the tissue shows HP1α and HP1β staining is dynamic, with HP1β at the foci periphery, localizing these two proteins to a distinct nuclear compartments. Scale bar: 50 μm. (**D**) IF staining intensity quantified from bottom panel of (**B**) shows HP1α (red) and HP1β (green) colocalization kinetics. A line was traced from the basal to the apical extent of the tissue and fluorescence intensity (gray values) were quantified. (**E**) Cut & Run signal tracks from chromosome 2 OR cluster. Values are reads per 10 million. Below the signal tracks, OR genes are depicted in different colors indicating the assigned expression zone. Antibodies used on IF: HP1α (ab109028) and HP1β (ab10811). Antibodies used for ChIP-seq H3K9me3 (Ab8898). Cut & Run HP1α (ab109028) and HP1β (D2F2, Cell Signaling, Danvers, MA 01923, USA). Apical and basal are used to indicate the outer and basal part of the tissue.

As OR alleles are chosen for expression in immature OSNs, the alleles that are not selected in a given neuron become enriched for H3K9me3 and recruited to a compact heterochromatic focus [29,34]. Given the co-localization of HP1β to these chromatin foci and the model for the function of this family of proteins in heterochromatin spreading [28], we sought to explore the role of HP1β in OR regulation. RNA-Seq levels of *Cbx1* (gene-encoding HP1β) and *Cbx5* (HP1α) from fluorescence-activated cell sorted (FACs) populations during OSN differentiation suggest they have a role in two distinct developmental windows (Figure 1A). *Cbx1* RNA expression peaks in the Mash1 (also known as *Ascl1*)-positive cells and Mash1/Neurogenin-1 double-positive cells, coincident with the step where stem cells commit to the neuronal lineage and undergo a dramatic reorganization of heterochromatin [12]. *Cbx1* RNA remains abundant in immature (Neurogenin1-positive and Atf5-translating populations) and mature olfactory sensory neurons (OMP-positive population). *Cbx5* RNA also peaks in Mash1-positive cells, when cells commit to the neuronal or sustentacular lineage; however, expression levels throughout differentiation are reduced compared to *Cbx1*, especially in cells that have committed to the neuronal lineage.

To visualize HP1α and HP1β cellular and subcellular protein localization in the olfactory epithelium, we performed Immunofluorescence (IF) (Figure 1B,C) and quantified fluorescence intensity (Figure 1D). HP1α staining is prominent throughout the nucleus in the basal cells of the olfactory epithelium, where stem cells reside, and in the sustentacular support cells which are most apical in the tissue. With differentiation, HP1α undergoes a dramatic change in localization, moving with the pericentromeric chromatin to the heterochromatic foci (Figure 1C) and gradually fades out from the system as olfactory neurons mature, both at the mRNA (Figure 1A) and protein levels (Figure 1B,C). On the other hand, HP1β shows staining throughout the neuronal layer (Figure 1B,C). Remarkably, in immature neurons, HP1β localizes to the heterochromatic foci and with maturation is found to encircle the heterochromatic foci of mature OSN (mOSN) in the MOE (Figure 1C,D; compare Hp1β localization in basal and apical cells). This staining pattern is consistent with olfactory receptor gene loci localization around the DAPI dense heterochromatic focus, as seen by DNA-FISH with a “pan-OR” probe, which is a probe with sequence homology to a substantial number of olfactory receptor genes [12]. Our RNA and protein expression analyses suggest a developmental switch or a sequential role for HP1 proteins during differentiation, except for early immature OSNs, where HP1α and HP1β both mark newly formed chromatin foci (Figure 1C). We propose independent roles for these proteins in OR gene regulation but do not discard a cooperative function in OR regulation and choice.

### 2.2. HP1β Is Enriched at OR Loci Compared to HP1α

Whether or not HP1α and HP1β have redundant roles in vivo has not been described. In vitro studies show HP1α can phase transition, but HP1β cannot [31,32]. The fact that the OR clusters migrate to the periphery of the chromatin foci in parallel with HP1β and that HP1α does not share compartment localization with HP1β, (Figure 1D) suggests a prominent role for HP1β in OR regulation and not HP1α. We predicted that HP1α would not interact with the OR gene clusters. To test this hypothesis, we performed Cut & Run using specific antibodies against HP1α and HP1β in pure populations of differentiated cells (mOSN) isolated by FACs (Figure 1E) and on micro-dissected tissue from zone-1 and zone-5 (Figure S1A). FRiP (Fraction of Reads in Peaks) score showed Cut & Run was successful (HP1α = 6.21% and HP1β = 12.45%). We observed high levels of HP1β protein incorporated into OR gene clusters compared to HP1α (Figure 1E and Figure S1). Peak analysis indicates HP1α and HP1β interact with the OR clusters in mOSN (Figure S1B and Figure S1C, but median count per million reads (median CPM) reveal that HP1α peaks in OR clusters are just above the background signal in contrast to HP1β which is enriched at these loci (Figure S1D). It is important to mention that these differences could be due to antibody efficiency, but in additional experiments in this study, we find that the HP1α and HP1β antibodies have similar efficiencies (see later). These results support our hypothesis that HP1 proteins have distinct functions in OR regulation with a major role for HP1β.

### 2.3. Constitutive Depletion of HP1β During Embryogenesis Leads to a Zonal Effect on OR Expression

**Figure 2:**
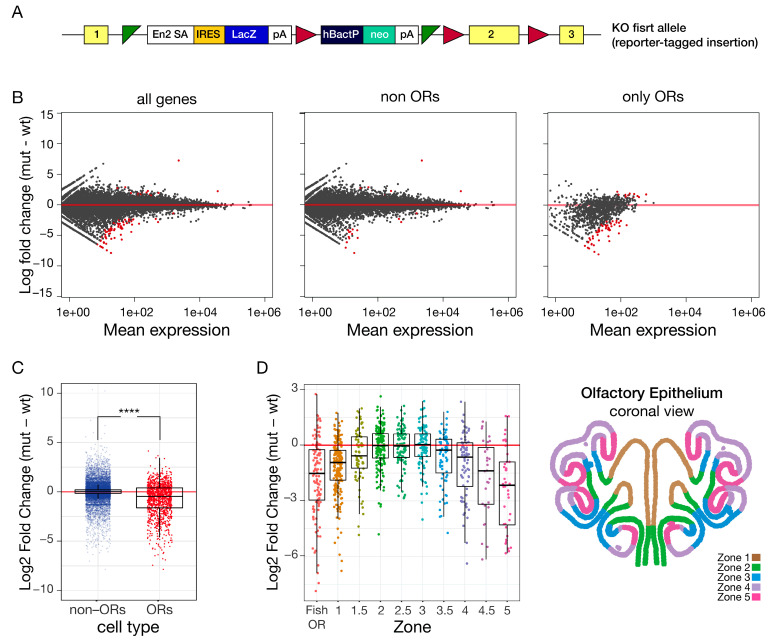
**OR expression is affected in a HP1β KO mouse model.** (**A**) Diagram of HP1β KO allele (from Sanger, Hinxton CB10 1SA, UK) used to study the dependence of olfactory choice regulation on HP1β. (**B**) RNA-seq analysis of gene expression in HP1β KO versus littermate controls. Bulk RNA-seq on E17.5 embryos. Significantly changed genes are colored red (*p*adj (Adjusted *p*-value) < 0.05 for greater than 1.5-Log2 Fold Change, Wald test, n = 3 biological replicates. (**C**) Log2 Fold change on bulk RNA-seq from E17.5 embryos comparing WT vs. HP1β KO versus littermate controls. Global Mean Log2FC = −0.7999. Global group *t*-test *p*-value 1.277 × 10^−210^ (****). (**D**) Boxplot for RNA-seq reads were bioinformatically separated into zones to which the OR receptors belong. Right, a diagram depicting the MOE divided into transcriptional zones is shown for the readers’ reference. Colors represent zones.

A previous study reported that HP1α constitutive knockout (KO) mice are viable with no obvious phenotype, while HP1β-constitutive KO mice die at birth or a few hours after [26]. This lethality could be due to anosmia, which would prevent pups from suckling, or due to genome instability [26]. To test the role of HP1β in OR regulation, we first turned to a knockout-first allele HP1β mouse from Sanger (Figure 2A), which exhibited embryonic lethality. We dissected the olfactory epithelium from Embryonic day (E)17.5 *Cbx1* KO embryos and their wildtype littermates. RNA-Seq from whole olfactory epithelium comparing *Cbx1* KO E17.5 embryos to control littermates showed reduced expression specifically for OR genes (Figure 2B). OR-genes and non-OR genes were considered separately for the purpose of the analysis. From 1194 OR genes receptors, 51 receptors (4.3%) showed a significant decrease in expression (adjusted *p*-value < 0.05 and |Log2 fold change| > 0.58)), and only 0.92% of OR genes showed a significant increase. In contrast, from 20,988 non-OR genes, only 15 genes (0.07%) were significantly downregulated, and 0.03% non-OR genes were significantly up-regulated. Interestingly, analysis of ORs shows a global reduction in expression in the HP1β KO mouse compared to littermate controls (mean Log2FC = −0.799 and a global group *p*-value—1.277 × 10^−210^) (Figure 2C). Constitutive loss of *Cbx1* causes a significant global reduction in OR-genes, with almost no change in non-OR genes (Figure 2B,C). This result supports HP1β’s role in olfactory receptor regulation, while raising questions about how loss of a repressive heterochromatin factor would repress gene expression. To further parse OR dysregulation in the *Cbx1* constitutive KO, the olfactory receptor genes were bioinformatically divided into anatomical zones, based on published data [36] (Figure 2D). Class I, or Fish ORs, the most evolutionarily ancient members of this gene family, which are anatomically expressed in zone-1, and zone-1 ORs exhibited reduced expression in the *Cbx1* KO, whereas zone-2 and zone-3 ORs were largely unaffected. Zone-4 and zone-5 ORs were expressed at greatly reduced levels compared to control littermates (Figure 2D). These data reveal a zonal effect on OR gene expression upon loss of HP1β.

### 2.4. Zone-4 and Zone-5 ORs Become Activated as Cbx1 and Cbx5 Switch During Development

HP1β constitutive knockout mice die in utero, at a stage when olfactory receptor expression is low compared to post-natal mice (Figure S2A). At birth, olfactory receptor expression increases up to five-fold compared to embryonic levels (Figure S2A,B). Interestingly, OR activation at birth is zonal (Figure S2C), with receptors from zone-4 and zone-5 exhibiting higher levels of activation compared to the other anatomical zones (while in embryos, zone-1 to zone-3 OR exhibited higher levels of expression compared to zone-4 and zone-5 ORs). This result shows that activation of the olfactory system does not happen synchronously. The developmental formation of this tissue could explain this asynchronous zonal activation, where the more interior tissues form first. Interestingly, *Cbx1* and *Cbx5* expression levels switch at birth, with *Cbx5* present at higher levels in E17.5 embryos and *Cbx1* becoming the dominant form after birth (Figure S2D,E). This result is concomitant with the activation of zone-4 and zone-5 ORs (Figure S2D,E) as OR differentiation and choice take place.

### 2.5. Ectopic HP1α Expression Partially Rescues High Lethality Observed in the HP1β KO Mice

**Figure 3:**
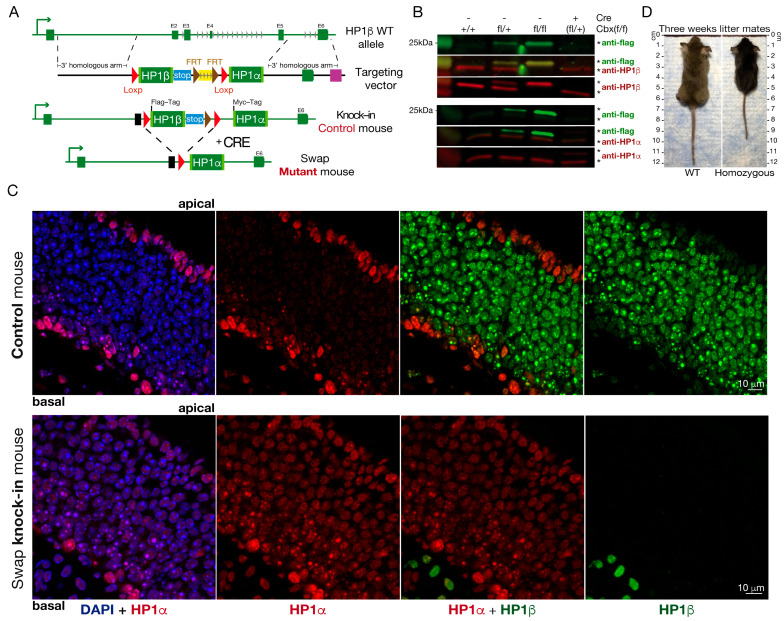
**HP1β swap by HP1α results in phenotypic defects and high lethality**. (**A**) Schematic representation of the swap mouse design. We targeted the HP1β locus with a construct containing the HP1β coding sequence, followed by a 3xStop codon and followed by HP1α coding sequence. After CRE recombination, HP1α will replace HP1β and the 3xStop codon removed. Hp1α knock-in will be regulated by HP1β endogenous promoter. (**B**) Western Blot on MOE protein extracts from WT (+/+), heterozygous (fl/+), and homozygous mice (fl/fl), with the specified antibodies. The predicted molecular weights are HP1α (22 kDa), myc- HP1α (~26 kDa), HP1β (26 kDa) and flag- HP1β (~29 kDa). The asterisk indicates cross-reactivity with the indicated antibody. (**C**) IF on HP1 knock-in (control) mouse (top panels) or after CRE recombination (bottom panels) using HP1α (red)- or HP1β (green)-specific antibodies. Antibodies used on WB: Anti-Flag (SIGMA M2 F-3165), HP1α (ab109028) and HP1β (ab10811). Antibodies used on IF: HP1α (ab109028) and HP1β (ab10811). (**D**) Picture from three weeks swap vs ctrl mouse littermates, shows a phenotypic effect on the size of the mutant mouse.

To determine whether HP1α (*Cbx5*) could rescue HP1β (*Cbx1*)-induced lethality, and gain deeper insight into how these proteins regulate OR choice and neuronal identity, we sought to generate a mouse in which the *Cbx1* coding sequence is replaced by *Cbx5*, placing HP1α under the native regulatory control of *Cbx1*. To accomplish this, we designed a conditional allele where *Cbx5* is regulated by the *Cbx1* endogenous promoter and *Cbx1* is knocked-out (Figure 3A). The first part of the inserted construct, which is flanked by two flox elements, has the coding sequence of *Cbx1* followed by 3X stop codons. The *Cbx5* coding sequence is downstream of the second flox element. We used the forebrain, neuron-specific *Foxg1*-Cre recombinase allele [37], to selectively delete *Cbx1* in neurons while simultaneously inducing expression of *Cbx5* under the control of the *Cbx1* endogenous promoter.

In the absence of Cre, the *Cbx1* knock-in is expressed from the endogenous *Cbx1* promoter, and the 3X stop prevents *Cbx5* from being expressed (Figure 3A,B, bottom panel). The Foxg1Cre allele [37] is a neuronal-specific recombinase that we use to conditionally delete *Cbx1* and “rescue” with *Cbx5* in neuronal lineages of the mouse, which includes the olfactory sensory neurons. Western blotting with an HP1α antibody shows that the transgenic HP1α band (Hp1α-myc tag, ~26 kDA) is present in *Cbx*^fl/+^; *Foxg1*-Cre mice, but absent in *Cbx*^fl/fl^ mice (referred to as control throughout) (Figure 3B, bottom panel). The band corresponding to the endogenous Hp1α protein (~22 kDa) is observed in both genotypes, as expected. This result demonstrates that in the absence of the recombinase Cre, the flag-HP1α protein is not expressed. In order to exclude the possibility that the knock-in construct changed *Cbx* RNA expression or HP1 protein levels, we performed Western blot (WB) and RNA-seq comparing WT versus the control mouse, and no significant differences were found (Figure 3B and Figure S3A,B). IF analysis shows similar HP1α and HP1β staining compared to WT in the *Cbx*^fl/fl^ control mouse (compare Figure 3C upper panel, with Figure 1B). Taken together, these data confirm that the knock-in control mouse is comparable to a WT mouse. We also determined that addition of HP1α in the *Cbx*^fl/+^; *Foxg1*-Cre heterozygous mouse does not lead to significant changes in protein levels between the endogenous and the introduced proteins (Figure 3B, right column).

After Cre recombination, Cre-positive progeny with two *Cbx* flox “HP1 swap” alleles (*Cbx*^fl/fl^; *Foxg1*-Cre, termed “swap mice” throughout) express HP1α wherever HP1β was expressed and do not express HP1β (Figure 3C, lower vs. upper panels). Upon Cre recombination, we rescue the lethal phenotype observed in the *Cbx1* KO (Figure 2); however, swap mice are runts and do not thrive as well as heterozygote or Cre-negative littermate controls. Few swap mice are viable until adult life, and most die between post-natal day (PN)0 and PN21. This reduced viability could be an effect of removing *Cbx1* or due to the ectopic expression of the inserted *Cbx5* gene. In support of the former, the heterozygous mice, *Cbx*^fl/+^, Cre-positive mice containing one copy of knocked-in HP1α and one copy of HP1β show no difference in viability when compared to *Cbx*^fl/fl^ control littermates, suggesting that phenotype is due to the absence of HP1β and not the exogenous expression of HP1α. As we used a neuronal-specific Cre, we cannot rule out the possibility that the reduced viability in swap mice is due to deficits in other neuronal systems.

IF in swap mice reveals that HP1β staining is absent from the MOE, highlighting successful knockout of the *Cbx1* gene (Figure 3C). We successfully induced *Cbx5* expression in swap mice, as exhibited by the strong staining of HP1α throughout the neuronal lineage of the olfactory epithelium, in stark contrast to a Cre-negative littermate control (Figure 3C). Importantly, this staining demonstrates that HP1α is now expressed in cells where endogenous HP1β was expressed; however, we do not observe HP1α staining encircling the DAPI dense foci where HP1β normally stains (Figure 3C). This result suggests differential recruitment of these two proteins to their targets, even if they are expressed in the same cell type. Our data demonstrate that under the control of the *Foxg1*-Cre recombinase, the *Cbx* swap construct successfully rescues peri-natal lethality observed in the *Cbx1* constitutive knockout, but *Cbx* swap mice exhibit a runt phenotype and reduced viability, pointing towards specific functions for HP1β protein in vivo.

### 2.6. OR Choice Is Disrupted in the Absence of HP1β

**Figure 4:**
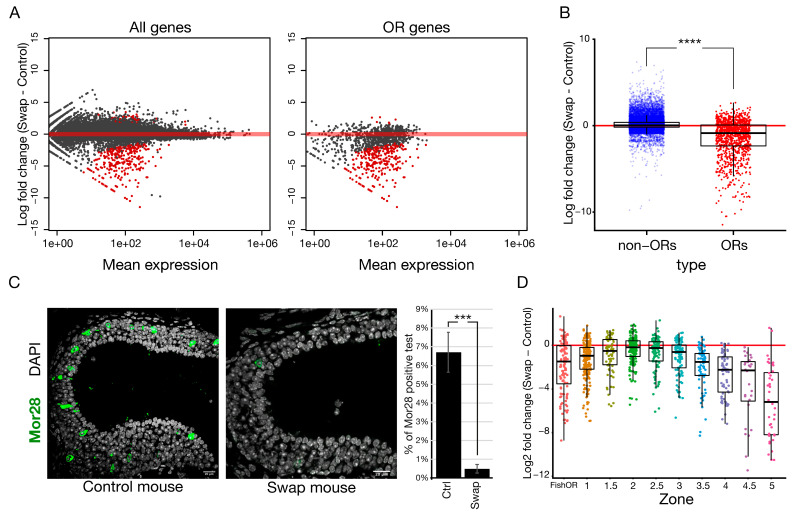
**HP1β has a specific role in OR gene regulation that HP1α cannot rescue.** (**A**) MA-plot of RNA-seq analysis of gene expression from sorted mOSN cells from swap mouse versus control mouse. Significantly changed genes are colored red (*p*adj (Adjusted *p*-value) < 0.05 for |Log2 Fold Change| > 0.58; Wald test; n = 3). (**B**) Log2 Fold Change analysis on samples used in (**A**). Swap mouse vs. control quantifying non-OR genes and OR genes. Global Mean Log2FC = −1.7562. Group *t*-test *p*-value: 3.8409 × 10^−92^ (****). (**C**) Left, IF with a zone-5 OR specific antibody (mor28, green) in zone-5 tissue sections, comparing control versus swap mouse. DAPI shown in gray. Scale bar is 20 μm. Right, quantification of percent Mor28 positive cells, n = 2 mice per genotype, 1148 WT cells vs. 997 Swap cells. Student’s *t*-test *p*-value = 0.001 (***). (**D**) Log2 Fold Change analysis on samples used from (**A**). RNA-seq reads were bioinformatically filtered into the zones to which the OR receptors belong. Colors represent zones.

To determine whether HP1α could rescue the effect in OR expression observed in the *Cbx1* KO mice (Figure 2A), we performed RNA-Seq in mOSN from the swap mice compared to littermate controls (Figure 4A). Our data show a dramatic down-regulation of 300 (25%) olfactory receptor genes (adjusted *p*-value < 0.05 with |Log2 fold change| > 0.58), with only 0.58% of the receptors showing a significant increase in expression. In contrast, non-OR genes were nearly unaffected, with six genes (0.028% of non-OR genes) exhibiting a significant decrease and 30 genes (0.14% of non-OR genes) significantly increased expression. Log2 fold change analysis comparing the swap mouse with control mice showed a significant effect on OR expression levels specifically (Figure 4B), with an observed OR group down-regulation of Log2FC = −1.75. *t*-test on the group Log2 fold change revealed a significant global shift (group *t*-test *p*-value = 3.8 × 10 × 10^−92^). We also confirmed this down-regulation at the protein level by IF (Figure 4C). The number of cells expressing the zone-5 OR Mor28 (*Olfr1507*) receptor was quantified in zone-5 olfactory epithelium tissue, showing a significant reduction in the number of OSNs that expresses this receptor compared to littermate controls (Figure 4C). To exclude the possibility that HP1α ectopic expression in the mature layer changes global gene expression, we performed RNA-seq comparing *Cbx*^fl/fl^ control littermates to heterozygous *Cbx*^fl/+^; *Foxg1*-Cre-positive mice, which contain one copy of the knocked-in *Cbx5* gene and one copy of the endogenous *Cbx1* gene (Figure S3C,D). We observe no change in gene expression when HP1α is ectopically expressed in OSN, strongly suggesting that loss of HP1β in the swap mice is what leads to changes in OR expression, and not ectopic HP1α expression. To determine whether HP1α can rescue zonal OR choice defects in *Cbx1* KO mice, we examined the zonal identities of dysregulated ORs in the swap mouse. We find that only zone-2 ORs were expressed at normal levels, comparable with RNA-Seq results from the *Cbx1* constitutive KO (Figure 2D). ORs from other zones showed decreased expression as a class, with zone-5 ORs exhibiting the strongest effect in expression (Figure 4D). These results show a zonal function for HP1β protein in OR regulation, with ORs from ventral zones being most strongly dysregulated. To explain this result, we considered different scenarios including cell death, cell maturation, skewed OR choice, or an effect on the transcriptional identity of the cell.

### 2.7. OR Choice and Zonal Identity Are Disrupted in the HP1 Swap Mice

**Figure 5:**
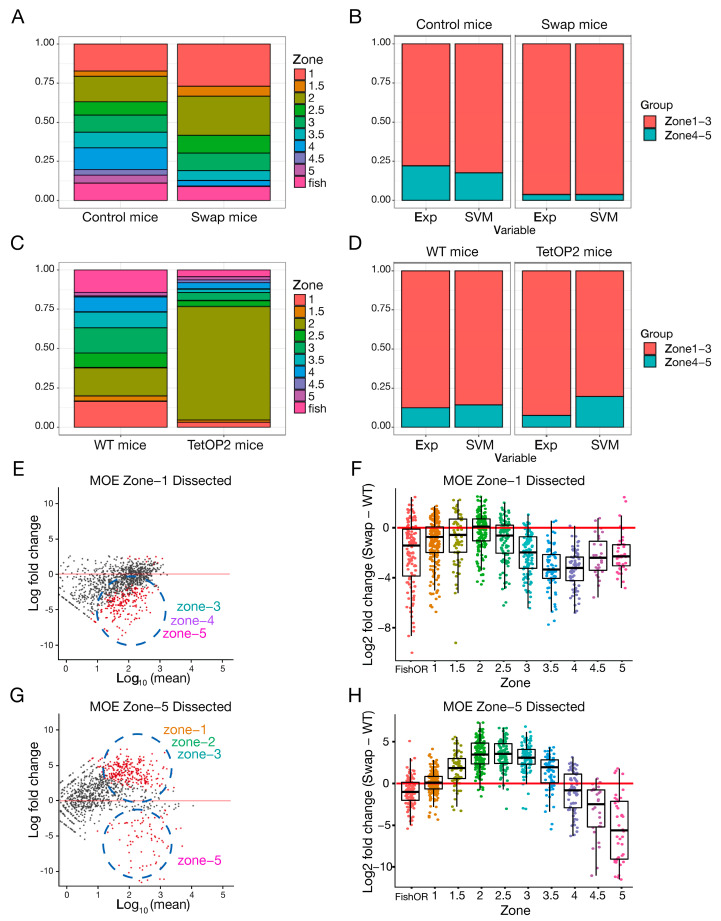
**HP1β regulates transcriptional identity and OR choice.** (**A**,**B**) Single Cell RNA-seq of mature OSNs from control mice vs. Swap mice: (**A**) stacked bar plot quantifying fraction of single cells that express an OR from a given zone. (**B**) In silico prediction analysis (using Support Vector Machine [38]) using transcriptional data from single cell RNA-seq. To train the model, we divided data from control mice in two groups: Cells expressing ORs from zones 1–3 and from zones 4–5 ((**B**), first column, experimental). Transcriptional differences (blind to chosen OR information) validate the model ((**B**), second column, SVM). Swap mouse analysis shows more OSNs express zone 1–3 ORs, accompanied by a change in zonal identity ((**B**), compare columns 3 and 4). (**C**,**D**) Single Cell RNA-seq from mature OSNs from *gg8tTA;tetO-P2* vs. *gg8tTA* control mice. The *gg8tTA* and *tetO-P2* alleles force P2 receptor expression, which is a zone 2 OR. (**C**) Stacked bar plot quantifying fraction of single cells expressing ORs from each zone in from *gg8tTA;tetO-P2* mice. (**D**) In silico prediction analysis. *gg8tTA;tetO-P2* mice express a zone-2 OR but cells maintain their original zonal identity. (**E**,**F**) RNA-seq analysis in physically micro-dissected cells from zone-1, mOSN-sorted cells. Gene expression analysis comparing swap mouse versus control mouse. Significantly changed genes are colored red (*p*adj < 0.05 and |Log2 Fold change| > 0.58, Wald test, n = 3). (**G**,**H**) RNA-seq analysis in physically micro dissected cells from zone-5, mOSN sorted cells. Significantly dysregulated genes are colored red (*p*adj < 0.05 and |Log2 Fold change| > 0.58, Wald test, n = 3). Cells from zone-5 show repression of zone-5 receptors and activation of zone-1 to zone-3 receptors. Colors represent zones.

Bulk RNA-Seq data demonstrate a dysregulation of OR genes in the swap mice. Since zone-5 epithelia is morphologically evident by imaging analysis, we eliminated cell death as an option and considered three possible explanations for these results: (1) OSNs from zone-4 and zone-5 are chosen but expressed at a lower level in each cell. (2) OR choice is skewed to zone-1 and zone-2 ORs. Downregulated ORs are chosen less frequently but zonal identity is not affected. (3) The neuronal identity of zone-4 and zone-5 has changed and cells physically located in these zones now express markers from other zones. To address these possibilities, we performed single-cell RNA-seq (scRNA-seq) comparing swap mice to littermate controls. We subset the cells to only consider mature OSNs. The chosen OR of each OSN was determined by the OR with the highest number of RNA counts (Figure 5A, control mice). Zonal identity of the chosen OR was assigned (Figure S4A,B). No difference in OR expression levels per cell was detected between the swap and control mice on a single-cell level (Figure S4C), suggesting that lower levels of zone-4 or zone-5 OR expression could not be explained by a decrease in expression per cell. In agreement with the skewed OR choice hypothesis, we observe zone-1 and zone-2 population expansion and a dramatic decrease in OSNs choosing ORs from zone-4 and zone-5 (Figure 5A, control vs. swap mice).

To determine whether the swap OSNs maintain their transcriptional identity of zone-4 and zone-5 OSNs, even if those OSNs chose a different OR, we determined the transcriptional differences between OSNs expressing ORs from dorsal zones-1 to zone-3 and ventral zone-4 to zone-5 (Figure 5B). To address this question, we analyzed our scRNA-seq data with the machine learning tool Support Vector Machine (SVM) [38] (Figure 5B). In the control mice, cells defined as mature OSNs were divided into zone-1 to zone-3 and zone-4 to zone-5 expressing cells based on the experimentally chosen ORs, and used to train the model (Figure 5B, control mice, experimental). Roughly 50 mRNAs in addition to the chosen ORs differ significantly between the two zonal groups. Some of these transcripts encode proteins involved in axon guidance which is in accordance with the hypothesis that one main function of zonal OR choice is to provide a first layer of organization for axon guidance to the olfactory bulb [39]. Next, sequencing reads corresponding to the chosen ORs were removed, and the algorithm was used to predict the zonal identity of the control OSN data set (Figure 5B, control mouse, experimental vs. SVM), blind to the expressed OR, validating the effectiveness of SVM zonal classification. In the swap mouse, SVM predicts that the ventral zone-4 to zone-5 OSNs have adopted a zone-1 to zone-3 identity (Figure 5B, swap mice, experimental vs. SVM), suggesting that these neurons are transcriptionally similar to zone-1 to zone-3 neurons and their zone-4 to zone-5 transcriptional identity has changed. This result contrasts with a second mouse model used as a negative control (Figure 5C,D) called *tetO-P2iGFP* (*tetOP2* [40]), where a *gg8tTA* driver specific to OSNs drives the expression of an inducible OR, the P2 allele. In *gg8tTA;tetO-P2* mice, OSNs are artificially biased to express the zone-2 OR *Olfr17* (P2) in the entire epithelium. SVM on a single-cell RNA-seq data set from these mice showed that while the majority of neurons have chosen a zone-2 OR (Figure 5C), the original transcriptional zonal identities remain intact (Figure 5D, *tetOP2* mice, experimental vs. SVM). This P2 mouse demonstrates that simply changing the expressed OR is not sufficient to change zonal transcriptional identity. Taken together, our data raise the exciting possibility that HP1β is required not only to ensure zonal OR choice but also to establish zonal transcriptional identity.

To experimentally confirm the SVM prediction, swap and littermate control olfactory epithelia were zonally micro-dissected to enrich zone-1 or zone-5 tissue (Figure 5E,G) as previously described [14]. RNA-seq was performed on pure populations of sorted mature OSNs using the OMP-ires-GFP allele, from these micro-dissected samples. As previously shown (Figure 4A), non-OR genes were nearly unaffected in micro-dissected mOSNs from swap mice, while a specific dysregulation of OR genes was observed (Figure 5E). Interestingly, zone-1 dissected tissue from swap mice (Figure 5E,F) exhibited a decrease in OR expression from zone-3 to zone-5, suggesting that in the WT context, HP1β facilitates expression of zone-3 to 5 ORs even in dorsal regions, and its absence in the swap further represses these ORs. Surprisingly, in zone-5 dissected tissue (Figure 5G,H), the swap mouse shows a drop in zone-5 ORs, and now exhibits an increase in the expression from zone-1 to zone-3 ORs compared to littermate controls. This increase in zone-1 to zone-3 ORs was masked when using RNA-seq from mOSN (Figure 4A), likely because ventral zone-5 tissue is a smaller proportion of the epithelium than larger dorsal zones. These results confirm our SVM prediction and show that olfactory sensory neurons physically located in zone-5 have chosen a different receptor, highlighting a role for HP1β in OR choice, specifically ensuring the full zonal repertoire of ORs. OR co-expression in the same cell was also examined with no clear differences between control and swap mice (Figure S4D). Taken together our single-cell and micro-dissected mOSN RNA-seq data suggest that HP1β regulates OR choice and the establishment of a transcriptional identity that cannot be rescued by HP1α.

### 2.8. HP1β-Dependent H3K9me3 Methylation Participates in OR Diversity

**Figure 6:**
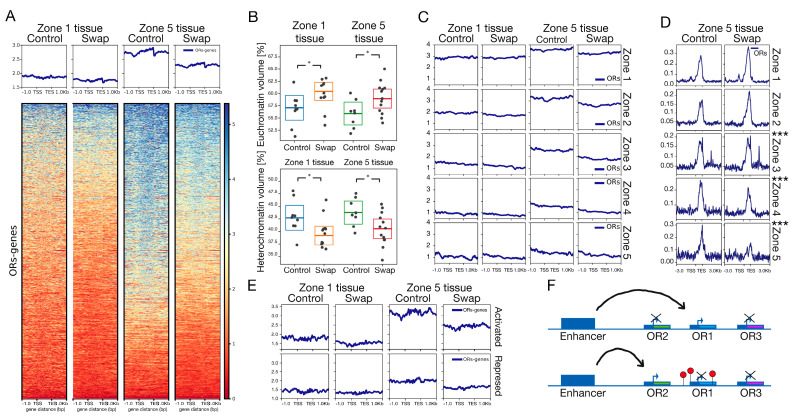
**HP1β is implicated in H3K9me3 incorporation and MOE zone formation.** (**A**) Native ChIP-seq on mature OSNs sorted from micro dissected tissue from zone-1 and zone-5. Swap versus control mouse shows loss of H3K9 methylation over OR clusters (n = 3 for each genotype). (**B**) Soft X-ray tomography on sorted OMP-GFP + mOSNs from micro dissected zone-1 and zone-5 tissue. Top plot shows percent of euchromatin volume (normalized by total nuclear volume) zone1-control (57%) vs. zone1-swap (60%), * *p* = 0.043, zone5-control (56%) vs. zone5-swap (59%), * *p* = 0.028. Bottom plot shows percent of heterochromatin volume (normalized by total nuclear volume) zone1-control (43%) vs. zone1-swap (40%), * *p* = 0.040, zone5-control (44%) vs. zone5-swap (40%), * *p* = 0.021. (**C**) Native ChIP-seq from FACs sorted mOSNs isolated from zone-1 and zone-5 micro dissected tissue. Reads were bioinformatically separated into zones. Zone-1 to zone-3 exhibit the greatest decrease in H3K9me3 in swap mice. This was observed only in zone-5 micro dissected and FACs sorted mOSN cells. (**D**) ATAC-seq on micro-dissected zone-1 vs. zone-5, ATAC-seq reads were bioinformatically separated into zones (n = 3 biological replicates each genotype). Difference between swap and control zone-5 samples was estimated as significant for zones-3 to zone-5, Wilcoxon test *p*-value < 0.001 (***) (zone-3 *p*-value: 1.4061 × 10^−04^, zone-4 *p*-value: 2.9558 × 10^−05^ and zone-5 *p*-value: 8.4903 × 10^−04^). (**E**) RNA-seq data sets (data from Figure 4) bioinformatically segregated into repressed and activated ORs and H3K9me3 levels of incorporation were determined. (**F**) Competition model proposes that OR receptors with a strong promoter will out-compete other receptors for their interaction with the enhancer hub. H3K9me3- HP1β dependent methylation will silence strong promoters, giving the opportunity to weaker promoters to be chosen for transactivation. Antibodies used for Native ChIP: H3K9me3 (Ab8898).

HP1β protein and Histone H3 lysine 9 (H3K9) lysine methylation are hallmarks of heterochromatin [20]. H3K9me3 incorporation into the OR genomic clusters occurs as OSNs in specific anatomical zones choose an OR for activation [14]. Given the zonal dysregulation of ORs in the swap mouse, and the gradual incorporation of H3K9me3 into the OR clusters during development [14], we hypothesized that H3K9me3 levels over OR loci could be zonally altered in the absence of HP1β. To test this, we performed Native Chromatin Immunoprecipitation (nChIP) using an antibody against H3K9me3 (Figure 6A). Consistent with a previous report [14], we observed differential levels of H3K9me3 incorporation in tissue between zone-1 and zone-5 (Figure 6A, control mice zone-1 vs. zone-5 tissue). With soft X-ray tomography (SXT), a technique that has the advantage of avoiding chemical fixation or nuclei isolation, we confirm heterochromatin levels are higher in mOSN from zone-5 vs. zone-1 (Figure 6B), supporting our nChIP observation (Figure 6A). In the absence of HP1β, total heterochromatin levels go down in comparison to control littermates (Figure 6B), as well as H3K9me3 levels over the OR loci (Figure 6A,C, swap mice). In agreement, SXT reveals that euchromatin levels go up in the absence of HP1β (Figure 6B). This result confirms that H3K9me3 levels are dependent on HP1β. To control for the specificity of this change, we examined the chromatin surrounding the OR enhancers, termed “Greek islands”, as they are regions of euchromatin immersed in heterochromatin. We observe no change in the H3K9me3 levels surrounding OR enhancers (Figure S5).

In order to determine what role H3K9me3 played in zonal OR regulation, we used micro-dissected tissue from zone-1 and zone-5 and bioinformatically segregated OR promoters into the five zones to assess H3K9me3 enrichment (Figure 6C). Remarkably, tissue from zone-5 shows a gradient of methylation, with zone-1 OR gene receptors (immersed in zone-5 tissue) having the highest levels of H3K9me3 incorporation and zone-5 the lowest (Figure 6C, zone-5, control column and Figure S6A,B). Our data provide evidence that H3K9me3 levels shape a repression gradient over ORs that determines zonal expression. In dorsal zone-1 tissue, low levels of H3K9me3 are observed over all ORs, and zone-1 ORs are expressed (Figure S6A,B, control zone-1 tissue). In ventral zone-5 tissue, zone-1 ORs harbor the highest H3K9me3 levels, followed by zone-2, with zone-5 ORs exhibiting the least amount (Figure S6A,B, zone-5 tissue), also coincident with the expression levels of zone-5 ORs. This result suggests a role for H3K9me3 in silencing the ORs in a zonal-specific manner.

If HP1β were required for the incorporation of H3K9me3 methylation gradients, we would expect to lose those gradients in the swap mouse, which is what we observe (Figure 6C, zone-5 swap mouse and Figure S6). We used ATAC-Seq to assess chromatin accessibility as a corollary to heterochromatin levels. In the absence of HP1β, we observed a gain in chromatin accessibility over zone-1 to zone-3 ORs, in agreement with lower H3K9me3 levels over OR loci described above (Figure 6D and Figure S5B). Zone-4 and zone-5 promoters in swap mice show a mild decrease in H3K9me3 levels (Figure 6C), but a significant decrease in chromatin accessibility (Figure 6D, bottom panels), coincident with a loss in activation as seen by RNA-seq (Figure 2B and Figure 4A). Previous reports demonstrate that transcription is sufficient to directly remodel the chromatin architecture of a gene [41,42]. In addition, in erythroid cells, it has been shown that losing activation by a gene’s corresponding enhancer was enough to lose transcription and chromatin accessibility at the transcriptional start site [30]. In the OR system, the receptors that are not activated by the enhancer hub become silenced [43]. Considering these previous reports, we predict zone-4 and zone-5 receptors lose chromatin accessibility as they lose the opportunity to be activated.

Different OSN populations in the MOE use distinct gene regulatory programs, so signals from small populations may be masked by those from more abundant cell populations in bulk tissue analyses. For this reason, we decided to examine histone methylation, considering activated vs. repressed OR genes (Figure 6E) as determined by RNA-seq (Figure 4A). We find that activated ORs show a greater reduction in methylation than repressed ORs in the swap mouse (Figure 6E), which results in homogenized methylation levels over the zones. This observation is even more evident when examining activated and repressed ORs in zonally micro-dissected tissue as methylation gradients are especially pronounced in WT zone-5 cells (Figure 6C and Figure S6A; zone-5; control vs. swap). Thus, in the absence of HP1β, ORs from all zones exhibit similar levels of H3K9me3 incorporation, losing the gradient of methylation observed in control mice. The molecular mechanism by which HP1β regulates the gradient distribution of H3K9me3 remains unclear. To test whether differential HP1β distribution throughout the MOE could explain the H3K9me3 deposition pattern observed (Figure 6C and Figure S6), we used two strategies. We examined the expression levels of *Cbx1* and *Cbx5* using RNA-Seq from micro-dissected zone-1 and zone-5 mOSN cells (Figure S7A). We found no zonal difference in RNA levels of these two genes. We also used single-cell RNA-seq to quantify *Cbx1* and *Cbx5* expression levels in neurons from all zones (Figure S7B,C), confirming no difference in *Cbx1* and *Cbx5* gene levels across the zones. Instead of tissue-specific expression of *Cbx* genes, our data instead support an enhancer competition model [44]. In this model, promoters compete to interact with enhancers. Strong promoters, as determined by upstream regulatory sequences, will out-compete weaker promoters, resulting in more frequent activation. Weaker promoters would depend on repression of stronger promoters to enable their expression (Figure 6F). In tissue from zone-1 MOE, low methylation levels favor the expression of stronger promoters. On the other hand, in tissue from zone-5, the establishment of gradients of methylation represses strong promoters, enabling “weaker” zone-5 promoters to be chosen.

Taken together, our data support a model where the promoter strengths [45] of dorsal ORs (zone-1 to zone-3) are able to “out-compete” ventral OR promoters. In tissue from zone-5 in a wildtype context, strong promoters (zone-1 to zone-3) will be silenced by H3K9me3, facilitating the activation of receptors from zone-4 and zone-5 and ensuring diversity in OR choice. We propose that losing the H3K9me3 repression gradient allows promoters from all zones to compete for their activation, resulting in activation of stronger promoters. This model of enhancer competition (Figure 6F) provides a framework to understand how the olfactory epithelium and potentially other tissues including neuronal systems similar to the olfactory system generate cellular diversity. Importantly, this model resolves the counterintuitive finding that both heterochromatin and OR expression are decreased in the swap mice. Loss of H3K9me3 gradients causes reduced expression of ventral zone ORs as dorsal ORs are ectopically activated.

### 2.9. HP1α Fails to Organize or Clusters Around the Heterochromatic Foci

**Figure 7:**
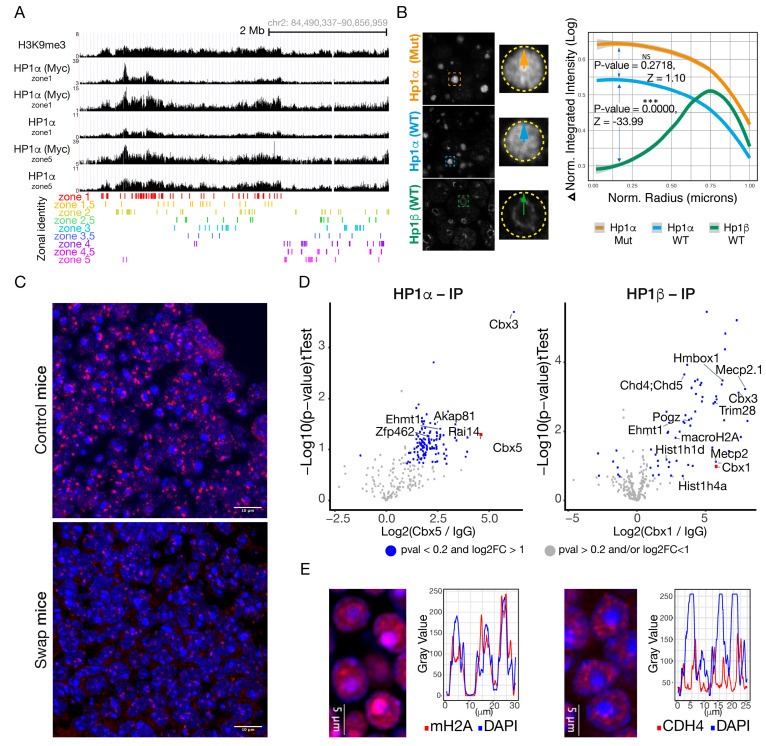
**HP1α fails to rescue HP1β functions**. (**A**) HP1α and HP1β Cut & Run signal tracks on mature sorted OSN from micro dissected zones. Chromosome 2 OR cluster is shown as a representative image. Values are reads per 10 million. Below the signal tracks, OR genes are depicted in different colors indicating the assigned expression zone. Results are shown in duplicates and with the use of different antibodies except for HP1β zone5. Antibodies used on ChIP: H3K9me3 (Ab8898) HP1α (HP1 alpha MA535397 ThermoFisher) and HP1β (D2F2, Cell Signaling). (**B**) Radial profile analysis for the proteins HP1α and HP1β in DAPI dense foci of the MOE. A circle was drawn around each focus, and the radial profile was measured. See Material and Methods for details. Fisher’s Z-test *p*-value < 0.001 (***), ns (no significant). (**C**) Representative image from DNA-FISH using a Pan-OR probe. The Pan-OR probe recognizes most of the OR-receptor sequences. Scale bar is 10 μm. (**D**) Mass Spectrometry of proteins immunoprecipitated from MOE tissue using an specific antibody against HP1α and HP1β. (**E**) IF imaging on MS candidates and staining intensity determination using FIJI and Intensity geomline. Scale bar is 5 μm.

If HP1α and HP1β are highly conserved proteins, why is HP1α unable to rescue the *Cbx1* KO phenotype? We envision three different scenarios to respond to this question. First, HP1α cannot interact with the OR clusters. Second, in the absence of HP1β, HP1α fails to segregate OR clusters from pericentromeric chromatin. Third, HP1α fails to recruit the enzymatic activities necessary for zonal OR choice. From RNA-seq, we know mature olfactory neurons have no *Cbx5* expression (Figure 1A) and this could explain the absence of HP1α observed on OR loci (Figure 1E). We decided to look at the swap mouse, where *Cbx5* expression is driven by the *Cbx1* promoter, forcing the ectopic expression of HP1α protein in mOSN. Surprisingly, with the use of Cut & Run, we find that HP1α is now enriched in OR gene loci in mOSN cells sorted from micro-dissected swap mouse tissue (Figure 7A). Profile heatmap around TSS of OR genes from Cut & Run counts show the difference in HP1α and HP1β recruitment (Figure S8A). The gradient blue-to-red color indicates high-to-low counts in the corresponding region and an increase in HP1α counts is observed in the swap mouse compared to controls. Median CPM confirms that this increase in HP1α recruitment in the swap mouse is significant compared to the control mouse (Figure S8B, Wilcoxon *p*-value = 4.481 × 10^−200^). It is important to emphasize that in the HP1α Cut & Run, the same antibody was used in both genetic backgrounds and experiments were performed in parallel. Peak analysis indicates HP1α interacts with the OR clusters, and share 222 peaks with H3K9me3 OR-specific peaks (Figure S8C). This result demonstrates that HP1α is capable of interacting with the OR clusters if ectopically expressed, but is not capable of regulating OR choice and identity as HP1β does in the WT context.

Interestingly, even after artificially expressing HP1α in mOSN, this protein fails to accumulate at the periphery of the chromatin foci (Figure 3C, bottom panel, Figure 7B and Figure S9). In the swap mouse, when comparing iOSN vs. mOSN, HP1α is localized to the heterochromatic foci, but only in the more basal part of the tissue (Figure 3C). In the more apical part, where mOSN are located, HP1α staining turns diffuse through the nucleus (Figure 3C). High-resolution microscopy (Figure S9) shows the presence of HP1α at the chromatin foci, but by visual inspection, HP1α staining seems to be homogenous and not to accumulate at the periphery of the foci (Figure 7B). With the use of radial profile analysis, we quantified the HP1α (WT), HP1β (WT), and HP1α knock-in (swap) protein localizations within the DAPI dense foci. This quantification unequivocally reveals that HP1α does not recapitulate HP1β localization around the periphery of the DAPI dense foci (Figure 7B). HP1α knock-in shows a similar pattern within the heterochromatic focus to HP1α WT (Figure 7B). Since HP1α can interact with the OR clusters (Figure 7A), these results suggest HP1α fails to organize the OR clusters into discrete OR compartments. To test this, we performed DNA-FISH using the Pan-OR probe (Figure 7C) which recognizes most of the olfactory receptor gene loci. Pan-OR signal was readily observed surrounding the heterochromatic foci in control mice, whereas in the swap mouse, Pan-OR fluorescence intensity signal was reduced, fewer puncta were observed per cell, and they were not organized around the heterochromatic foci. This result suggests either HP1β or the balance between HP1α and Hp1β is necessary to organize the ORs around the heterochromatin foci.

We hypothesized that the protein-interacting partners of these two Hp1 proteins could explain HP1α’s inability to recruit H3K9me3 to OR clusters. With the use of Mass Spectrometry (MS), we determined the protein interactors for HP1α and HP1β (Figure 7D and Figure S10A). We performed immunopurification with antibodies against HP1α, HP1β and a control IgG using whole MOE lysate. Pearson correlation shows high correlation between replicates and distinct separation between IP targets. Gene set enrichment analysis (GSEA) shows significant enrichment of proteins involved in histone binding, histone deacetylase binding, among others, for both proteins (Figure S10A). Amongst the top candidates for HP1β (cutoff pval < 0.05 and log2FC > 2), we identified previously reported interactors, including Trim28 [46], Ehmt1 [47,48,49], macroH2A [48], and Chd4 [50]. Some of these candidates are of particular interest including Ehmt1, a histone methyltransferase involved in H3K9me2 incorporation [47,48]. The Trim family of proteins has previously been shown to recruit HP1 proteins to diverse systems [50,51,52]. With the use of IF, dependent on the availability and quality of antibodies, we confirmed the co-localization of selected MS candidates in HP1β compartments (Figure 7E and Figure S10B). Interestingly, macroH2A was identified as an HP1β interactor, which fits with the observation that its incorporation into chromatin happens in parallel with HP1β recruitment as OSNs differentiate (Figure 7E and Figure S10B). Remarkably, macroH2A staining colocalizes with the DAPI dense foci (Figure 7E), but it is also enriched in a halo around the chromatin foci, as we observe for HP1β in mOSNs (Figure 1B). MacroH2A does not interact with HP1α by Mass Spectrometry (Figure 7E). The Chromodomain–Helicase-DNA-Binding Protein 4 (Chd4) stains discrete domains around the DAPI dense foci (Figure 7E). Chd4 is an ATP-dependent helicase that binds and distorts nucleosomal DNA [52,53], and acts as a component of the histone deacetylase NuRD complex which is involved in chromatin remodeling and repression [52,54,55,56]. Chd4 enrichment in chromatin foci paired with its deacetylase activities coincide with the gain of repressive chromatin on the OR foci [12]. The nuclear corepressor for KRAB domain-containing zinc finger proteins (KRAB-ZFPs, also known as Trim28) is known for mediating transcriptional repression by recruiting repressive activities [56,57]. Trim28 staining in the MOE is diffused through the epithelium (Figure S10B) but stains around the nuclear foci in mature OSNs (Figure S10B). Finally, it is interesting that the two HP1 isoforms (α and β) interact with HP1γ by IP-MS (Figure 7D). HP1γ, contrary to HP1α and β, localizes to larger stretches of euchromatin [58] and is significantly enriched at promoters of active genes [59]. HP1γ may play important roles in cell fate decisions in stem cells [60] and in mesodermal lineage decisions as its knockdown inhibits neural differentiation [61,62]. Since HP1γ also interacts with H3K9me2/3, it will be interesting to analyze its role in OSN diversity and identity. In conclusion, HP1β enrichment in the chromatin foci is accompanied by chromatin remodeling proteins that likely facilitate the further incorporation of repressive chromatin and segregation of OR gene loci into discrete compartments around the DAPI dense focus. These data lead us to propose that HP1β but not HP1α, through its interaction with the OR foci and specific protein interactors, recruits chromatin remodeling complexes, and facilitates H3K9me3 incorporation into the OR clusters.

## 3. Discussion

**Figure 8:**
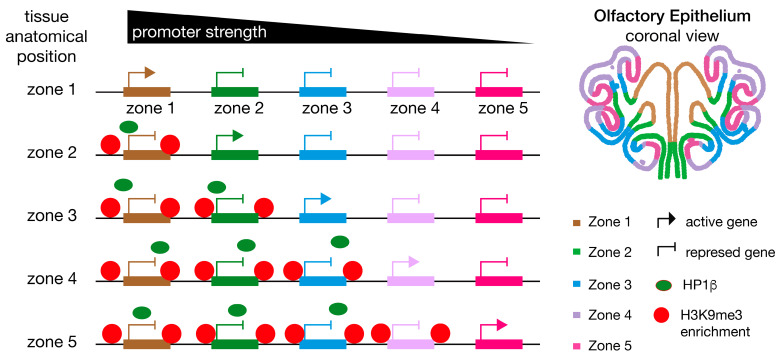
**Model for OR receptor gene choice and establishment of zonal identity**. Working model. H3K9 methylation is incorporated at different levels in the mouse MOE. H3K9me3 shows gradients of repression that decorate the 5 anatomical zones. We suggest methylation will silence stronger promoter compared to the receptors expressed in the corresponding zone, allowing weaker receptors to be chosen. The recruitment of HP1β ensures chromatin remodeling activities and repression.

With this work, we reveal a function for HP1β in OR gene choice and neuronal diversity. Our swap mouse design allowed us to test the function of HP1 proteins in vivo, enabling imaging and biochemical assays in the context of neuronal differentiation and cellular identity acquisition. Importantly, we uncovered a mechanism by which a stochastic process becomes biased. Based on our data presented here and previous work on OR gene enhancer regulation [11,16,17,43], we propose a model for ORs gene choice and the establishment of zonal identity that depends on the competition of OR promoters to interact with the OR enhancer hub (Figure 8). We propose that this competition is mediated by the incorporation of H3K9me3 in an HP1β-dependent manner (Figure 6A–C). Our model proposes that the HP1β-dependent incorporation of H3K9me3 creates repression gradients, blocking strong promoters from expression in more ventral zones. The competition of promoters for enhancers would depend on the promoter strength defined by their sequence composition [63]. In dorsal zones from the MOE, where low levels of H3K9me3 were found (Figure 6A,C), we propose that strong promoters (zone-1 ORs) will interact with the OR enhancer hub and become active. In ventral zones from the MOE, where receptors from zone-5 have the propensity to be chosen, receptors from zone-1 to zone-4 acquire higher levels of histone methylation (Figure 6A–C), enabling weaker promoters (zone-5 ORs) to be chosen. In the swap mouse, loss of H3K9me3 over OR loci (Figure 6) causes OSNs in more ventral zone 5 tissue to adopt a dorsal identity and express dorsal ORs (Figure 4 and Figure 5). In our model, this phenotype is explained by the stronger promoters of dorsal ORs, which out-compete ventral ORs in the context of incomplete heterochromatinization.

Monoallelic regulation of OR expression has been explained by two main models. The stochastic model proposes that the same set of transcription factors (TFs) participate in the stochastic selection of one allele [64]. The deterministic model predicts that specific combinations of *cis*-elements and transcription factors are required to express a gene within a defined spatial zone [65]. In support of the deterministic model, diverse OR gene promoter structures have been described, including promoters with or without Ebf binding sites, and with or without TATA-box elements [66,67], which have functional consequences on OR expression [68]. In addition, in a recent publication, a specific score for each OR was determined that defines the spatial location at which the specific receptor is expressed [63]. The DV score depends on the genomic sequences located upstream of each receptor and on the level of heterochromatinization [63]. We support the coexistence of the two models: a deterministic mechanism founded on a range of regulatory sequences that will generate a repertoire of promoters with different strengths (similar to the DV score) and a stochastic mechanism that will stochastically choose among promoters having a similar strength. Within this framework, our data suggest that histone H3K9 methylation is key to silencing strong promoters, giving weaker promoters a chance to be chosen.

### 3.1. HP1β Has a Specific Role in OR Choice That HP1α Cannot Rescue

HP1β protein was found to colocalize with the heterochromatic chromatin foci [12] and as an H3K9me3 interactor [56], with roles in diverse processes including chromatin formation [29] and gene silencing [57]. With the use of IF and RNA-Seq (Figure 1), we showed the dynamics of HP1α and HP1β expression and localization through OSN differentiation, OR choice, and neuronal maturation. HP1α expression decreases early during differentiation and OR choice, suggesting either an early function for this protein or a specific role for HP1β. Interestingly, by IF (Figure 1B), we observe HP1α and HP1β occupy different chromatin compartments: HP1α stains the DAPI dense foci while HP1β stains the OR compartments. We report a developmental window shared by these two proteins that suggests they may be working in a sequential manner or together at a very early step to set up the nuclear architecture distinctive of the mature olfactory sensory neurons. We also observe HP1α interaction with the OR clusters in the swap mouse (Figure 7A), revealing that HP1α is capable of interacting with the OR loci. In the WT context, HP1β interaction with OR loci (Figure 1E) may inhibit or dissolve HP1α from the OR clusters, as has been reported in an in vitro model [69]. We predict that the interplay between HP1 paralogs may be necessary to setup heterochromatin over OR genes in early stages of differentiation, but further studies are required to determine the role, if any, of HP1α and HP1γ proteins in OR choice.

By using an HP1β constitutive KO model, we found HP1β is required for expression of the full repertoire of ORs (Figure 2). Our swap model system confirms these findings by RNA-Seq (Figure 4A) and by using a specific antibody against an OR from zone-5 (Figure 4C). We show that *Cbx5* knock-in (HP1α) expression rescues the high lethality observed in the *Cbx1* KO; however, OR-specific phenotypes are not rescued by Hp1α. Taken together, we show for the first time in vivo that HP1β has specific functions in transcriptional regulation that HP1α cannot rescue.

### 3.2. HP1β Regulates OR Gene Choice and Transcriptional Identity

The HP1β KO mice and the swap mice showed skewed OR expression to receptors from zones-1 to zone-3. ORs from ventral zones were less likely to be chosen in both genetic contexts (Figure 2D and Figure 4D). Single-cell RNA-seq confirms that fewer OSNs choose zone-4 or zone-5 ORs (Figure 5A). Comparative expression analysis shows no decrease in OR transcription levels per cell (Figure S4C), supporting an effect related to OR choice instead of OR transcriptional levels. In silico analysis from single-cell RNA-seq data (Figure 5B) and RNA-seq on micro-dissected tissue from zone-1 and zone-5 OSNs (Figure 5E–H) showed cells have instead chosen a receptor from a different zone. The transcriptional identity of zone-5 OSNs is skewed to zone-1 to zone-3 transcriptional identities, revealing changes in global transcription patterns that extend beyond the chosen OR (Figure 5G,H). Ectopic expression of zone-2 P2 receptor throughout the entire olfactory epithelium showed no effect on transcriptional identity, highlighting an independent role for HP1β in establishing zonal transcriptional identity (Figure 5C,D). These results demonstrate that the absence of HP1β affects OR choice and cell identity, as receptors from dorsal zones of the MOE have colonized the tissue.

### 3.3. HP1β Regulates OR Choice by a Mechanism That Includes H3K9me3

The HP1 family of proteins is known for its participation in chromatin formation and gene silencing through mechanisms that involve histone methylation [8]. In the swap mouse, H3K9me3 levels are reduced globally including over OR gene clusters (Figure 6 and Figure S6). Curiously, the effect on H3K9me3 is not homogenous: dorsal ORs that incorporate heterochromatin first (described in [14]) exhibit a more severe reduction in H3K9me3. This result supports H3K9me3-HP1β-dependent incorporation in ORs via a regulated mechanism to be refined in future studies. Our data in the MOE support the generally accepted mechanism to explain the spreading of heterochromatin [28]. The histone H3 methylation levels reported in GBC cells [19] are likely responsible for recruiting members of the HP1 family to OR clusters; subsequently, HP1β will recruit additional histone methyltransferases and chromatin remodeling complexes including G9a and the NuRD complex identified in this work as an HP1β interactor (Figure 7D), which in turn facilitate the incorporation of H3K9me3 to block ORs with stronger promoters. We find that H3K9me3 forms gradients of repression in tissue from zone-5 and not from zone-1 (Figure 6C and Figure S6), resulting in distinct heterochromatin patterns in different zones. This study supports a role for H3K9me3 in restricting the repertoire of receptors from which each neuron can choose, enabling the expression from the 1300 ORs in a zonal fashion that ensures cell diversity in the MOE.

### 3.4. Cooperative and Unique Roles of HP1 Proteins

The HP1 family of proteins was first described to play a central role in gene silencing [21,23]. Extensive sequence homology within conserved domains of the three paralogs suggests redundant functions (reviewed in [22]), but domain-swapping experiments reveal unique properties of Hp1 family members [70]. This can be explained by small differences in their DNA sequence, which promote the recruitment of distinct interacting partners with their enzymatic activities [71], as well as differences in DNA and/or chromatin affinity [69,72], in their nuclear localization [58,73,74,75,76], and in their biophysical properties [31,32]. The clearest example of how small differences in the DNA sequence can confer unique properties is observed with the N-terminal domain of HP1α, which allows this protein to experience in vitro liquid–liquid phase separation (LLPS) [31,32]. HP1α also promotes condensates in H3K9me3-containing arrays [77], and is the sole paralog that can form stable HP1α-DNA condensates [69]. Interestingly, while HP1β is unable to form LLPS [31,32], it readily dissolves HP1α-DNA condensates in vitro [69]. To date, in vivo LLPS studies have been difficult to interpret due to complex interactions in biological systems [78] but the olfactory system presents an attractive model to assay for LLPS properties of HP1 paralogs, and the interplay among them.

HP1 proteins participate in a variety of regulatory processes including elongation, sister chromatid cohesion, chromosome segregation, telomere maintenance, DNA repair, and RNA splicing (reviewed in [79]). Unraveling the unique and cooperative functions of each member of this family is imperative to understanding the chromatin dynamics and HP1 protein roles in transcriptional regulation. During cell differentiation, HP1β, but not HP1α, is involved in the maintenance of the pluripotency state [27], but in differentiated cells, HP1β has the opposite effect: it disrupts the maintenance of the differentiated state and facilitates cell reprogramming [27]. HP1-deficient nematodes reveal overlapping and distinct roles for HP1 paralogs in DNA repair [80]. Genome instability and defects in neuronal development were observed in neurons derived from *Cbx1* knockout mice [26]. In contrast, neurons derived from *Cbx5*-deficient mice show no phenotype [26]. We find that HP1α and HP1β migrate to different compartments in neurons of the olfactory system (Figure 1). RNA-seq and scRNAseq on micro-dissections prove non-redundant functions for HP1α and HP1β in cellular identity and OR choice (Figure 3D and Figure 4). In the absence of HP1β in the olfactory system, HP1α rescues the embryonic lethality observed by us (Figure 3) and others [26], but OR choice and neuronal transcriptional identity are compromised (Figure 5). This result shows in vivo that HP1 proteins have specific functions that cannot be rescued by other HP1 paralogs. With the use of the mouse model generated in this study, future work could elucidate the function of these proteins in other systems in vivo.

Protein subcellular localization provides insight into protein function [81]. In interphase, HP1α and HP1β localize to heterochromatin [58,73,75], including centromeres and telomeres, [82]. HP1γ, in contrast, associates with euchromatic regions [73,74,75,76] and actively transcribed genes [83]. In human and mouse cell lines, HP1α and HP1β are enriched at the pericentric heterochromatin (PCH) [84], with a clear role in heterochromatin formation [85]. HP1γ is also present at PCH, but its interaction depends on the combined existence of the other two isoforms [84]. In our IP-MS experiment, we found that HP1γ interacts with HP1α and HP1β (Figure 7D). It would be interesting to determine if HP1γ is recruited to ORs and PCH in OSN, and if so, whether that depends on the presence of HP1α and HP1β proteins. This could clarify principles of chromatin and nuclear organization in the OR system, especially to understand if the HP1 paralogs have unique or cooperative functions.

### 3.5. HP1α Fails to Generate a Diverse Olfactory Epithelium

We propose a role for HP1β in the process of H3K9me3 incorporation or maintenance of the repressive state, which is necessary to promote tissue diversity. It is also important, however, to understand why the conserved family member HP1α fails to promote tissue diversity. Cut & Run experiments show that HP1α can interact with the OR gene loci in the swap mouse, but this interaction is not sufficient to incorporate high levels of H3K9 methylation (Figure 7A), as observed in the WT context. HP1 proteins can work as an anchor for other proteins. We identify specific chromatin regulatory proteins as interactors of HP1β but not HP1α, including the Chd4 NURD member, which is a chromatin remodeling complex involved in transcriptional silencing, as well as members of the Trim family, with scaffold proteins involved in gene silencing that work by recruiting epigenetic machinery to repress gene expression. We found that Ehmt1, a histone methyl transferase, interacts with both HP1 proteins. While additional work is required to confirm this part of the model, our data suggest that through its protein network, HP1β has a greater potential to participate in targeted OR silencing than HP1α.

From our work and work from other labs, we suggest that HP1β and, possibly, HP1α have roles in organizing the OR chromatin foci. In vitro studies have recently shown that HP1β can form liquid droplets in the presence of H3K9me3 [86,87], which may explain the formation of the OR compartments around the nuclear foci decorated with HP1β and H3K9me3 in OSNs (Figure 7B,C). The differential incorporation of H3K9me3 over OR clusters and the homologs’ different properties of phase transition likely facilitate the segregation of OR compartments in the nucleus. In this work, we propose a role for HP1β in incorporating gradients of H3K9me3, which serve to partition the MOE in zones with distinct chromatin architecture patterns that ensure OR choice from the complete repertoire of ORs. We demonstrate a specific role for HP1β and not HP1α in OR choice and transcriptional identity formation.

## 4. Materials and Methods

### 4.1. Mouse Strains

Mouse protocols were approved by the Columbia University IACUC under protocol number AC-AAAT2450 and AC-AABG6553. All mice were housed in standard conditions with a 12 h light/dark cycle and access to food (regular diet) and water ad libitum. Mice were sacrificed using CO_2_ followed by cervical dislocation. *Cbx1* KO mice are from EUCOMM, Sanger (strain name: EPD0027_2_B01). Gene: *Cbx1*; colony prefix: MAAT; ESC clone ID: EPD0027_2_B01; allele: *Cbx1^tm1a(EUCOMM)Wtsi^*; allele type: Knockout First, Reporter-tagged insertion with conditional potential. *Cbx* swap mouse was custom designed and made by Biocitytogen (Regeneron Pharmaceuticals); Gene: *Cbx1*; allele: *Cbx1(Cbx5)* CKI; model design: *Cbx1*^tm1a(EUCOMM)Wtsi^. The targeting vector containing the construct LoxP, flag-*Cbx1*, STOP, myc-*Cbx5*, LoxP was inserted in the *Cbx1* locus by homologous recombination, eliminating exons 2 to 5 from the endogenous *Cbx1* coding sequence (gene ID: 12412 (NCBI)). Mice with induced expression of the P2 receptor were obtained by crossing tetO-P2-IRES-GFP [40] mice to *Gng8(gg8)-tTA* mice ([2]). Mature OSNs were isolated by breeding in the OMP-ires-GFP allele, which marks OMP+, mOSNs with GFP [64].

### 4.2. Zonal Dissection of the Olfactory Epithelium

Fluorescent signal in *Olfr545*-delete-YFP (zone 1 OR), *Olfr17*-ires-GFP (zone 2 OR), and *Olfr1507*-ires-GFP [16] (zone 5 OR) mice were used to standardize dorsal (zones-1) MOE and ventral (zone 4–5) MOE micro-dissections (See [14]). The accuracy of dissections was confirmed by RNA-seq.

### 4.3. Immunofluorescence

MOE was dissected and frozen in OCT compound (Fisher, Waltham, MA 02451, USA, Catalog No. 23-730-571) and sectioned at 12 µm with a cryostat (Leica CM1950, Heidelberger Str. 17–19, 69226 Nussloch, Germany) onto a microscope slide (Fisher Scientific). Tissue sections were dried at RT for ten minutes, fixed with 4% PFA, 1X phosphate-buffered saline (PBS) for 5 min, washed with 1X PBS, then blocked for 30–60 min in blocking solution (4% Sterile Donkey Serum (Sigma, D9663-10ML, St. Louis, MO 63103, USA), 1% Triton X-100, 1X PBS). Slides were incubated in a humid chamber overnight with primary antibody diluted 1:200 in blocking solution, with a coverslip placed on top to avoid evaporation. The following day, coverslips were removed by adding 1X PBS to the top, slides were washed in a slide mailer 3 × 5 min in PBST (1X PBS, 0.1% Triton X-100), then incubated in blocking solution with secondary antibody (diluted 1:500) and DAPI (diluted 1:1000) before mounting with Vectashield mounting media (Vector Labs, H-1900-2, Newark, CA 94560, USA). Images were acquired using a Zeiss LSM 700 Confocal (7344 Oberkochen, Germany), a W1-Yokogawa spinning disk Confocal, or a Zeiss LSM 800 series Confocal microscope with Airyscan, depending on experiment.

### 4.4. Immunofluorescence Analysis

All analyses for images acquired after immunofluorescence were completed in FIJI (version 2.16.0/1.54p) and R studio (Version 2025.09.2+418). *Average Signal Intensity*: a line was traced with the “Straight Segment” tool through the MOE from basal to apical. Fluorescence Intensity Profile was analyzed and the gray value was extracted. Data was saved as .csv document and plotted using R.

### 4.5. Fluorescence-Activated Cell Sorting

Cells from the MOE were treated with papain for 40 min at 37 °C according to the Worthington Papain dissociation protocol. Cells were washed 2X with cold PBS 1X, passed through a 40 μm strainer filter, and sorted on a Beckman Coulter MoFlo Astrios EQ Cell Sorter. Fluorescence compensation was not performed because all fluorochromes of interest utilized different laser sources and had clearly separated emission spectra. Sheath pressure was set at 28 PSI and sample pressure was kept below 28.5 PSI. A 100 μm nozzle was used and event rates were between 5000 and 15,000 cells per second. For cell sorting experiments, a droplet drive frequency of 49.6 kHz was used, cells were sorted in “purify” mode and the drop envelope was “1–2”. Gating was performed to isolate cells, remove doublets (using the area, height and width side scatter measurements from the 488 laser), and remove DAPI+ dead cells. Laser and detector settings were as reported in Table 1.

For RNA-seq and native ChIP-seq, DAPI-negative (live cells) and fluorescent cells (OMP-GFP+) were collected.

### 4.6. DNA FISH

Fresh mouse olfactory epithelia were embedded in Optimal Cutting Temperature compound (OCT—Sakura) and frozen to −20 °C. Cryosections were cut at 6 µm, air-dried for at least 30 min and fixed in ice-cold 4% PFA for 5 min. Fixation was stopped by rinsing the sections two times with permeabilization media PBS 1% Triton (PBST). DNA was fragmented with 0.1M hydrochloric acid (HCl) for 15 min at room temp (RT), digested with 3 mg/mL of RNase A (Thermo Scientific™, Waltham, MA 02451, USA, Catalog No. 10753721) in PBST for 1 h at 37 °C and dehydrated by 3X 70%/90%/100% ethanol washes at 45 °C for 5 min. Sections were denatured at 85 °C for 5 min in 2× SSC, 75% formamide (Invitrogen), and immediately dehydrated as before but with ethanol at ice-cold temperature. PanOR probe (Biotin-Nick Translation Mix, Roche, 4070 Basel, Switzerland, Cat. Number 11745824910) was added at 25 ng/µL, covered with 8 mm circular coverslips, sealed with rubber cement and incubated ON at 37 °C. Slides were washed in 2× SSC, 55% formamide, 0.1% NP-40 3X for 15 min, rinsed in PBST, blocked in TNB (Promega TSA kit, Madison, WI 53711, USA), and incubated 2 h RT with anti-dig conjugated to DyLight fluors (Jackson Immunoresearch, West Grove, PA, USA), 3X washed in PBST + 8% formamide and mounted. Images were collected on a confocal Zeiss LSM700 microscope.

### 4.7. RNA-Seq and Analysis

Mouse olfactory epithelia were dissected and lysed or FAC-sorted mOSNs were lysed using Trizol with standard protocols in an RNA clean area. DNase treatment was performed with Turbo DNA-free kit (Ambion, Austin, TX 78744, USA, Cat. Number AM1907), sequencing libraries were prepared with the Illumina TruSeq Stranded Total RNA library kit (San Diego, CA 92122, USA), QC was determined with Agilent Bioanalyzer (Santa Clara, CA 95051, USA), and libraries were sequenced on a NextSeq550 or HiSeq2500 Illumina sequencer. Cut adapt was used to remove adapter sequences and reads were aligned to the mm10 genome with STAR (version: 2.7.11b) [88]. Samtools (Version: 0.1.19-96b5f2294a) [89] was used to select high-quality reads (−q.30). Differential expression analysis was performed in R with DESeq2 (version: v1.26.0–1.30.0) [90]. For all RNA-seq data, *p*-values refer to adjusted *p*-value (*p*adj) calculated in DESeq2 with tests specified in figure legends. Genes were considered significantly dysregulated if *p*-value < 0.05 and |Log2 Fold Change| > 0.58. Fragments per kilobase of transcript per million mapped read; FPKM were converted to TPM and used to analyze Log2 fold change in zonal-OR genes.

### 4.8. Native Chromatin Immunoprecipitation

Native ChIP was performed as described in Magklara et al. [19]. Briefly, nuclei were isolated: FACS-sorted cells were pelleted (600× *g*, 10 min, 4 °C) and resuspended in ice-cold Buffer I (0.3 M Sucrose, 60 mM KCl, 15 mM NaCl, 5 mM MgCl_2_, 0.1 mM EGTA, 15 mM Tris-HCl pH 7.5, 0.1 mM PMSF, 0.5 mM DTT, 1× protease inhibitors). Cells were lysed by adding an equal volume of ice-cold Buffer II (Buffer I + 0.4% NP-40) and incubating on ice for 10 min. Nuclei were pelleted (1000× *g*, 10 min, 4 °C) and resuspended in 250 µL of ice-cold MNase buffer (0.32 M Sucrose, 4 mM MgCl_2_, 1 mM CaCl_2_, 50 mM Tris-HCl pH 7.5, 0.1 mM PMSF, 1× protease inhibitors). Micrococcal Nuclease (MNase) Digestion: 0.1 U of MNase (Sigma) per 100 µL of MNase buffer was added, and the mixture was incubated for 1 min and 40 s in a 37 °C water bath. Digestion was stopped by adding EDTA to a final concentration of 20 mM. Chromatin Fractionation: The first soluble chromatin fraction (S1) was pelleted and supernatant was stored overnight at 4 °C. The undigested material (pelleted) was resuspended in 250 µL of ice-cold dialysis buffer (1 mM Tris-HCl pH 7.5, 0.2 mM EDTA, 0.1 mM PMSF, 1× protease inhibitors) and rotated overnight at 4 °C. The undigested material was pelleted by centrifugation and constitutes the S2 fraction. S1 and S2 fractions were combined and 5% of the combined chromatin was saved as input. Immunoprecipitation (IP): Chromatin was diluted to 1 mL in Wash Buffer 1 (50 mM Tris-HCl pH 7.5, 10 mM EDTA, 125 mM NaCl, 0.1% Tween-20, 5× protease inhibitors) and rotated overnight at 4 °C with 1 µg of antibody. Dynabeads (10 µL Protein A and 10 µL Protein G per IP) were pre-blocked overnight at 4 °C with 2 mg/mL yeast tRNA and 2 mg/mL BSA in Wash Buffer 1. Blocked beads were washed once with Wash Buffer 1, then added to the antibody-bound chromatin and rotated for 2–3 h at 4 °C. Beads were washed 4× with Wash Buffer 1, 3× with Wash Buffer 2 (50 mM Tris-HCl pH 7.5, 10 mM EDTA, 175 mM NaCl, 0.1% NP-40, 1× protease inhibitors), and 1× with TE buffer (pH 7.5). Elution and DNA Purification: Immunoprecipitated DNA was eluted by resuspending the beads in 100 µL of Native ChIP Elution Buffer (10 mM Tris-HCl pH 7.5, 1 mM EDTA, 1% SDS, 0.1 M NaHCO3) using a thermomixer (37 °C, 900 rpm, 15 min). This elution step was repeated twice, and the eluates were combined. Library Preparation and Sequencing: Sequencing libraries were prepared using the NuGEN Ovation V2 DNA-Seq Library Preparation Kit (San Carlos, CA 94070, USA). Sequencing was performed using 50 bp paired-end (PE) reads on a HiSeq 2500 or 75 bp PE reads on a NextSeq 550.

### 4.9. Cut & Run

Mouse olfactory epithelia were micro-dissected for zone 1 or zone 5 tissue. Mature OSNs from zonal micro-dissections were isolated by FAC sorting OMP-GFP-positive cells from swap and control mice as described above. Each batch for each zone was centrifuged and resuspended in 1× PBS. CUT&RUN was performed using the CUTANA CUT&RUN Kit (Epicypher, Durham, NC 27709, USA, 14-1048) according to the manufacture protocol.

### 4.10. ATAC-Seq

ATAC-seq was performed from live sorted cells using the protocol developed by the Buenrostro lab [91]. Briefly, cells were pelleted and then resuspended in lysis buffer (10 mM Tris-HCl, pH 7.4, 10 mM NaCl, 3 mM MgCl_2_, 0.1% IGEPAL CA-630). Nuclei were immediately pelleted (1000 rcf, 10 min, 4 °C). Pelleted nuclei were resuspended in transposition reaction mix prepared from Illumina Nextera reagents (for 50 µL: 22.5 µL water, 25 µL 2 × TD buffer, 2.5 µL Tn5 Transposase). The volume of the Tn5 transposition reaction was scaled to the number of cells collected: 1 µL mix per 1000 cells. If fewer than 10,000 cells were collected by FACS, 10 µL reactions were performed. Transposed DNA was column purified using a Qiagen MinElute PCR cleanup kit (Qiagen, 40724 Hilden, Germany). The transposed DNA was then amplified using barcoded primers and NEBNext High Fidelity 2× PCR MasterMix (NEB, Ipswich, MA 01938-2723, USA). Amplified libraries were purified using Ampure XP beads (Beckman Coulter, Brea, CA 92821-6232, USA) at a ratio of 1.6 µL of beads per 1 µL of library and eluted in 30 µL of elution buffer (10 mM Tris-HCl pH 8, 0.1 mM EDTA).

### 4.11. Native ChIP-Seq and ATAC-Seq Analysis

Adapter sequences were removed with Cut Adapt. ChIP-seq and ATAC-seq reads were aligned to the mouse genome (mm10) using Bowtie2 [92]. Default settings were used, but a maximum insert size of 1000 (−X 1000) was allowed for ATAC-seq and native ChIP-seq data since these data sets contained some large fragments. PCR duplicate reads were identified with Picard and removed with Samtools [89]. Samtools was used to select uniquely aligning reads by removing reads with alignment quality alignments below 30 (−q 30). HOMER v4.11 [93] was used to call peaks of ChIP-seq signal using the ‘factor’ mode and an input control. Consensus peak sets were generated by selecting peaks that overlapped between biological replicates and extending them to their combined size. For signal tracks, replicate experiments were merged, and HOMER was used to generate 1 bp resolution signal tracks normalized to a library size of 10,000,000 reads. For H3K9me3 ChIP-seq, replicate experiments were merged, and HOMER was used to generate 1 bp resolution signal tracks normalized to a library size of 10,000,000 reads. Regions enriched for H3K9me3 were identified by running HOMER peak calling.

### 4.12. Single-Cell RNA-Seq in Olfactory Lineage Cell Types

Fresh mouse olfactory epithelia were dissected and dissociated with papain for 40 min (Worthington Biochemical, Lakewood, NJ, USA) at 37 °C according to the Worthington Papain Dissociation System. Cells were washed 2× with cold PBS before passing through a 40 μm strainer. Cells were counted and processed according to 10X Genomics manufacturer instructions using 10X Genomics equipment and reagents from the Chromium Single-Cell Gene Expression v3 kit (6230 Stoneridge Mall Road, Pleasanton, CA 94588, USA). Samples were processed with the help of the Columbia Genome Center. Libraries were sequenced on an Illumina NextSeq 550 Sequencing machine, and sequencing data were aligned with Cell Ranger (10X Genomics) with introns included. Data were analyzed with Seurat V4 [94] and R studio. Experiment was performed with two biological replicates.

### 4.13. SXT Sample Preparation

Zonally dissected individual mOSNs were collected by FACs sorting OMP-GFP-positive cells from swap and control mice as described above. Each batch for each zone was centrifuged and resuspended in 1× PBS. Single cells were prepared following the protocol described in [57]. In brief, cells were loaded in 8 µm wide glass capillaries and cryopreserved after plunge-freezing in liquid nitrogen-cooled liquid propane. Samples were then stored in liquid nitrogen prior to data acquisition.

### 4.14. SXT Data Collection

SXT was performed at the National Center for X-Ray Tomography (ncxt.org) on the beam line 2.1, located at Lawrence Berkeley National Laboratory, as presented in [57]. X-ray projection images were collected at an energy of 517 eV using a 50 nm resolution objective lens. During the data acquisition, samples were cryopreserved in a stream of liquid nitrogen-cooled helium. A total of 92 projection images were sequentially captured at 2° increments around a 180° axis of rotation (capillary axis), with an exposure time of 300 ms. Cells located in areas of the capillary with diameter larger than 15 µm were collected using the half acquisition protocol, as presented in [95]. A total of 184 projection images were sequentially captured at 2° increments around a 360° axis of rotation, with an exposure [96] time of 350 ms. Each tomogram was reconstructed using the algorithm AREC3D [96]. To calculate the linear absorption coefficient (LAC), each pixel intensity was normalized as presented in [96].

### 4.15. Tomogram Segmentation

The segmentation of cellular structures was performed using semiautomated methods and visualized and refined in the Amira 2021.2. software. The cell membrane was initially segmented using the algorithm ACSeg 3D [97] on the Biomedisa (https://biomedisa.info/) server [98]. The nuclear envelope was manually segmented using the function “paintbrush” in Amira 2021.2.

### 4.16. Partitioning Nucleus into Hetero-/Eu-Chromatin

To segment the reconstructed linear absorption coefficient (LAC) of the nucleus into hetero and euchromatin, we used a smoothed Gaussian mixture modeling (GMM) approach. Using the manually segmented nucleus as a mask, a two-component GMM [99] was fit to the (mean) normalized LAC values. The model’s posterior probabilities were interpolated back into the volume and smoothed. The final segmentation was obtained by thresholding the smoothed probabilities at 0.5. Based on previous observation provided by Le Gros et al., 2016 [100], mature olfactory neurons were selected based on the total amount of heterochromatin being above 37% of the total nuclear volume [101].

### 4.17. Splitting Heterochromatin into Inner and Outer Compartments

Using a watershed approach, we segmented heterochromatin into inner and outer regions [102]. First, a thin border region along the nuclear envelope (two voxels thick) was used to extract the characteristic local thickness [103] of the peripheral heterochromatin. Seeds for the watershed segmentation were selected based on their distance to the nuclear envelope: voxels within the 50th percentile of the local thickness formed the outer seed, and voxels beyond the 90th percentile of the local thickness formed the inner seed. We removed small regions (less than 1% of the largest) from the inner seeds.

Using these seeds, we performed watershed segmentation on the Euclidean distance transform of the heterochromatin mask to partition the heterochromatin into distinct inner and outer compartments.

### 4.18. Affinity Purification Mass Spectrometry

Cells were lysed in lysis buffer (150 mM NaCl, 50 mM Tris pH 7.5, 1% IGPAL-CA-630 Sigma #18896, 5% glycerol) on ice for 20 min, clarified by centrifugation, and total protein was quantified by BSA quantification. A total of 5 ug antibody was added to 1ug total protein lysate per replicate. The cell lysates with antibody were incubated with magnetic beads overnight at 4 °C. Supernatants were removed, beads were washed two times with wash buffer plus NP-40 (50 mM Tris pH 7.5, 150 mM NaCl, 5% glycerol, 0.05% NP-40), and twice with wash buffer (50 mM Tris pH 7.5, 150 mM NaCl, 5% glycerol). After the last wash, the beads were resuspended in 80 μL trypsin buffer (2 M Urea, 50 mM Tris pH 7.5, 1 mM DTT, 5 μg/mL trypsin) to digest the bound proteins at 25 °C for 1 h at 1200 rpm. The supernatant was collected and the beads were washed twice with 60 μL Urea buffer (2 M Urea, 50 mM Tris pH 7.5) and the washes were combined with the supernatant. The combined elution was cleared of residual beads by a quick spin. A total of 80uL of the elution was used and disulfide bonds were reduced with 5 mM dithiothreitol (DTT); cysteines were subsequently alkylated with 10 mM iodoacetamide. Samples were further digested by adding 0.5 μg sequencing grade-modified trypsin (Promega) at 25 °C. After 16 h of digestion, samples were acidified with 1% formic acid (final concentration). Tryptic peptides were desalted on C18 StageTips according to [104], evaporated to dryness in a vacuum concentrator, and reconstituted in 15 μL of 3% acetonitrile/2% formic acid for LC-MS/MS.

LC-MS/MS analysis was performed on a Q-Exactive HF. A total of 5 μL of total peptides was analyzed on a Waters M-Class UPLC using a 15 cm Ion-Optics column (1.7 μm, C18, 75 μm × 15 cm) coupled to a benchtop ThermoFisher Scientific Orbitrap Q Exactive HF mass spectrometer. Peptides were separated at a flow rate of 400 nL/min with a 90 min gradient, including sample loading and column equilibration times. Data were acquired in data-dependent mode. MS1 spectra were measured with a resolution of 120,000, an AGC target of 3e6 and a mass range from 300 to 1800 *m*/*z*. MS2 spectra were measured with a resolution of 15,000, an AGC target of 1 × 10^5^ and a mass range from 200 to 2000 *m*/*z*. MS2 isolation windows of 1.6 *m*/*z* were measured with a normalized collision energy of 25.

Proteomics raw data was analyzed by MaxQuant v2.0.3.0 [104] using a UniProt database (Mus musculus, UP000000589), and MS/MS searches were performed under default settings with LFQ quantification. Data was further analyzed in R v3.6.3. Contaminants and proteins only identified by site or reverse were removed; a pseudocount randomly taken from the bottom of the signal distributions was added to the LFQ intensity values and then the LFQ intensity values were log2 transformed. Proteins with a mean MS/MS count value for each IP condition below five were removed from subsequent analysis. Interacting proteins were identified as those that passed a log2FC sample IP over control IP cutoff of 1 and a *p*-value of 0.2.

## Figures and Tables

**Table 1:** Fluorochrome used for cell sorting. Laser wavelength, lase power, band-pass filter, PMT and gain are indicated for each fluorochrome.

Fluorochrome	Laser Wavelength (nm)	Laser Power (mW)	Band-Pass Filter	PMT (V)	Gain
DAPI	405	80	448/59	350	1
GFP	488	200	513/26	399	2
tdtomato	561	200	579/16	335	3

## Data Availability

The original contributions presented in this study are included in the article/Supplementary Materials. Further information and requests for resources and reagents should be directed to the lead contact Martin Escamilla: martin.escamilla13@gmail.com. Raw genomic data are available at Cut & Run (release date 23 March 2026): https://www.ncbi.nlm.nih.gov/geo/query/acc.cgi?acc=GSE324835; ATAC-Seq (release date 23 March 2026): https://www.ncbi.nlm.nih.gov/geo/query/acc.cgi?acc=GSE324834; scRNA-seq (release date 23 March 2026): https://www.ncbi.nlm.nih.gov/geo/query/acc.cgi?acc=GSE324728; ChIP-seq (release date 23 March 2026): https://www.ncbi.nlm.nih.gov/geo/query/acc.cgi?acc= GSE324864; RNA-seq: (release date 23 March 2026): https://www.ncbi.nlm.nih.gov/geo/query/acc.cgi?acc= GSE325634; Proteomics (release date 23 March 2026): ftp://massive-ftp.ucsd.edu/v12/MSV000101119/.

## Supplementary Materials

The following supporting information can be downloaded at https://www.mdpi.com/article/10.3390/ijms27072958/s1.

## References

[1] Malissen M., Trucy J., Jouvin-Marche E., Cazenave P.-A., Scollay R., Malissen B. (1992). Regulation of TCR α and β gene allelic exclusion during T-cell development. Immunol. Today.

[2] Nguyen M.Q., Zhou Z., Marks C.A., Ryba N.J.P., Belluscio L. (2007). Prominent roles for odorant receptor coding sequences in allelic exclusion. Cell.

[3] Augui S., Nora E.P., Heard E. (2011). Regulation of X-chromosome inactivation by the X-inactivation centre. Nat. Rev. Genet..

[4] Chaumeil J., Skok J.A. (2013). A new take on v(d)j recombination: Transcription driven nuclear and chromatin reorganization in rag-mediated cleavage. Front. Immunol..

[5] Chess A., Simon I., Cedar H., Axel R. (1994). Allelic inactivation regulates olfactory receptor gene expression. Cell.

[6] Ferguson-Smith A.C. (2011). Genomic imprinting: The emergence of an epigenetic paradigm. Nat. Rev. Genet..

[7] Rister J., Desplan C. (2011). The retinal mosaics of opsin expression in invertebrates and vertebrates. Dev. Neurobiol..

[8] Tasic B., Nabholz C.E., Baldwin K.K., Kim Y., Rueckert E.H., Ribich S.A., Cramer P., Wu Q., Axel R., Maniatis T. (2002). Promoter choice determines splice site selection in protocadherin alpha and gamma pre-mRNA splicing. Mol. Cell.

[9] Wojtowicz W.M., Flanagan J.J., Millard S.S., Zipursky S.L., Clemens J.C. (2004). Alternative splicing of Drosophila Dscam generates axon guidance receptors that exhibit isoform-specific homophilic binding. Cell.

[10] Kanata E., Duffié R., Schulz E.G. (2024). Establishment and maintenance of random monoallelic expression. Development.

[11] Monahan K., Lomvardas S. (2015). Monoallelic expression of olfactory receptors. Annu. Rev. Cell Dev. Biol..

[12] Clowney E.J., LeGros M.A., Mosley C.P., Clowney F.G., Markenskoff-Papadimitriou E.C., Myllys M., Barnea G., Larabell C.A., Lomvardas S. (2012). Nuclear aggregation of olfactory receptor genes governs their monogenic expression. Cell.

[13] Lyons D.B., Allen W.E., Goh T., Tsai L., Barnea G., Lomvardas S. (2013). An epigenetic trap stabilizes singular olfactory receptor expression. Cell.

[14] Bashkirova E.V., Klimpert N., Monahan K., Campbell C.E., Osinski J., Tan L., Schieren I., Pourmorady A., Stecky B., Barnea G. (2023). Opposing, spatially-determined epigenetic forces impose restrictions on stochastic olfactory receptor choice. eLife.

[15] Monahan K., Schieren I., Cheung J., Mumbey-Wafula A., Monuki E.S., Lomvardas S. (2017). Cooperative interactions enable singular olfactory receptor expression in mouse olfactory neurons. eLife.

[16] Monahan K., Horta A., Lomvardas S. (2019). LHX2- and LDB1-mediated trans interactions regulate olfactory receptor choice. Nature.

[17] Pourmorady A.D., Bashkirova E.V., Chiariello A.M., Belagzhal H., Kodra A., Duffié R., Kahiapo J., Monahan K., Pulupa J., Schieren I. (2024). RNA-mediated symmetry breaking enables singular olfactory receptor choice. Nature.

[18] Lyons D.B., Magklara A., Goh T., Sampath S.C., Schaefer A., Schotta G., Lomvardas S. (2014). Heterochromatin-mediated gene silencing facilitates the diversification of olfactory neurons. Cell Rep..

[19] Magklara A., Yen A., Colquitt B.M., Clowney E.J., Allen W., Markenscoff-Papadimitriou E., Evans Z.A., Kheradpour P., Mountoufaris G., Carey C. (2011). An epigenetic signature for monoallelic olfactory receptor expression. Cell.

[20] Bannister A.J., Zegerman P., Partridge J.F., Miska E.A., Thomas J.O., Allshire R.C., Kouzarides T. (2001). Selective recognition of methylated lysine 9 on histone H3 by the HP1 chromo domain. Nature.

[21] Eissenberg J.C., James T.C., Foster-Hartnett D.M., Hartnett T., Ngan V., Elgin S.C. (1990). Mutation in a heterochromatin-specific chromosomal protein is associated with suppression of position-effect variegation in Drosophila melanogaster. Proc. Natl. Acad. Sci. USA.

[22] Canzio D., Larson A., Narlikar G.J. (2014). Mechanisms of functional promiscuity by HP1 proteins. Trends Cell Biol..

[23] James T.C., Elgin S.C. (1986). Identification of a nonhistone chromosomal protein associated with heterochromatin in Drosophila melanogaster and its gene. Mol. Cell. Biol..

[24] Casale A.M., Cappucci U., Piacentini L. (2021). Unravelling HP1 functions: Post-transcriptional regulation of stem cell fate. Chromosoma.

[25] Vakoc C.R., Mandat S.A., Olenchock B.A., Blobel G.A. (2005). Histone H3 lysine 9 methylation and HP1gamma are associated with transcription elongation through mammalian chromatin. Mol. Cell.

[26] Aucott R., Bullwinkel J., Yu Y., Shi W., Billur M., Brown J.P., Menzel U., Kioussis D., Wang G., Reisert I. (2008). HP1-beta is required for development of the cerebral neocortex and neuromuscular junctions. J. Cell Biol..

[27] Mattout A., Aaronson Y., Sailaja B.S., Raghu Ram E.V., Harikumar A., Mallm J.-P., Sim K.H., Nissim-Rafinia M., Supper E., Singh P.B. (2015). Heterochromatin Protein 1β (HP1β) has distinct functions and distinct nuclear distribution in pluripotent versus differentiated cells. Genome Biol..

[28] Jenuwein T. (2001). Re-SET-ting heterochromatin by histone methyltransferases. Trends Cell Biol..

[29] Becker J.S., Nicetto D., Zaret K.S. (2016). H3K9me3-Dependent Heterochromatin: Barrier to Cell Fate Changes. Trends Genet..

[30] Escamilla-Del-Arenal M., Recillas-Targa F. (2008). GATA-1 modulates the chromatin structure and activity of the chicken alpha-globin 3′ enhancer. Mol. Cell. Biol..

[31] Larson A.G., Elnatan D., Keenen M.M., Trnka M.J., Johnston J.B., Burlingame A.L., Agard D.A., Redding S., Narlikar G.J. (2017). Liquid droplet formation by HP1α suggests a role for phase separation in heterochromatin. Nature.

[32] Strom A.R., Emelyanov A.V., Mir M., Fyodorov D.V., Darzacq X., Karpen G.H. (2017). Phase separation drives heterochromatin domain formation. Nature.

[33] Sanulli S., Trnka M.J., Dharmarajan V., Tibble R.W., Pascal B.D., Burlingame A.L., Griffin P.R., Gross J.D., Narlikar G.J. (2019). HP1 reshapes nucleosome core to promote phase separation of heterochromatin. Nature.

[34] Cvetkovska V., Hibbert A.D., Emran F., Chen B.E. (2013). Overexpression of Down syndrome cell adhesion molecule impairs precise synaptic targeting. Nat. Neurosci..

[35] Duffié R., Shayya H., Escamilla Del Arenal M., Wang M., Kahiapo J., Ugurbil A., Keskin A., Clowney F., Bashkirova E.V., Pourmorady A.D. (2025). Mex3a-dependent post-transcriptional silencing ensures olfactory receptor diversity and axon guidance specificity. Cell Rep..

[36] Tan L., Xie X.S. (2018). A Near-Complete Spatial Map of Olfactory Receptors in the Mouse Main Olfactory Epithelium. Chem. Senses.

[37] Hébert J.M., McConnell S.K. (2000). Targeting of cre to the Foxg1 (BF-1) locus mediates loxP recombination in the telencephalon and other developing head structures. Dev. Biol..

[38] Cortes C., Vapnik V. (1995). Support-vector networks. Mach. Learn..

[39] Ressler K.J., Sullivan S.L., Buck L.B. (1993). A zonal organization of odorant receptor gene expression in the olfactory epithelium. Cell.

[40] Fleischmann A., Abdus-Saboor I., Sayed A., Shykind B. (2013). Functional interrogation of an odorant receptor locus reveals multiple axes of transcriptional regulation. PLoS Biol..

[41] Chahar S., Ben Zouari Y., Salari H., Kobi D., Maroquenne M., Erb C., Molitor A.M., Mossler A., Karasu N., Jost D. (2023). Transcription induces context-dependent remodeling of chromatin architecture during differentiation. PLoS Biol..

[42] Stewart-Morgan K.R., Reverón-Gómez N., Groth A. (2019). Transcription Restart Establishes Chromatin Accessibility after DNA Replication. Mol. Cell.

[43] Markenscoff-Papadimitriou E., Allen W.E., Colquitt B.M., Goh T., Murphy K.K., Monahan K., Mosley C.P., Ahituv N., Lomvardas S. (2014). Enhancer interaction networks as a means for singular olfactory receptor expression. Cell.

[44] Nordin M., Bergman D., Halje M., Engström W., Ward A. (2014). Epigenetic regulation of the Igf2/H19 gene cluster. Cell Prolif..

[45] Plessy C., Pascarella G., Bertin N., Akalin A., Carrieri C., Vassalli A., Lazarevic D., Severin J., Vlachouli C., Simone R. (2012). Promoter architecture of mouse olfactory receptor genes. Genome Res..

[46] Groner A.C., Meylan S., Ciuffi A., Zangger N., Ambrosini G., Dénervaud N., Bucher P., Trono D. (2010). KRAB-zinc finger proteins and KAP1 can mediate long-range transcriptional repression through heterochromatin spreading. PLoS Genet..

[47] Liu H., Wang W., Weng X., Chen H., Chen Z., Du Y., Liu X., Wang L. (2021). The H3K9 histone methyltransferase G9a modulates renal ischemia reperfusion injury by targeting Sirt1. Free Radic. Biol. Med..

[48] Hoyer-Fender S., Czirr E., Radde R., Turner J.M.A., Mahadevaiah S.K., Pehrson J.R., Burgoyne P.S. (2004). Localisation of histone macroH2A1.2 to the XY-body is not a response to the presence of asynapsed chromosome axes. J. Cell Sci..

[49] Chin H.G., Estève P.-O., Pradhan M., Benner J., Patnaik D., Carey M.F., Pradhan S. (2007). Automethylation of G9a and its implication in wider substrate specificity and HP1 binding. Nucleic Acids Res..

[50] Sun F., Yang Q., Weng W., Zhang Y., Yu Y., Hong A., Ji Y., Pan Q. (2013). Chd4 and associated proteins function as corepressors of Sox9 expression during BMP-2-induced chondrogenesis. J. Bone Miner. Res..

[51] Maksakova I.A., Thompson P.J., Goyal P., Jones S.J., Singh P.B., Karimi M.M., Lorincz M.C. (2013). Distinct roles of KAP1, HP1 and G9a/GLP in silencing of the two-cell-specific retrotransposon MERVL in mouse ES cells. Epigenet. Chromatin.

[52] Hoffmeister H., Fuchs A., Erdel F., Pinz S., Gröbner-Ferreira R., Bruckmann A., Deutzmann R., Schwartz U., Maldonado R., Huber C. (2017). CHD3 and CHD4 form distinct NuRD complexes with different yet overlapping functionality. Nucleic Acids Res..

[53] Farnung L., Ochmann M., Cramer P. (2020). Nucleosome-CHD4 chromatin remodeler structure maps human disease mutations. eLife.

[54] Sillibourne J.E., Delaval B., Redick S., Sinha M., Doxsey S.J. (2007). Chromatin remodeling proteins interact with pericentrin to regulate centrosome integrity. Mol. Biol. Cell.

[55] Tong J.K., Hassig C.A., Schnitzler G.R., Kingston R.E., Schreiber S.L. (1998). Chromatin deacetylation by an ATP-dependent nucleosome remodelling complex. Nature.

[56] Lachner M., O’Carroll D., Rea S., Mechtler K., Jenuwein T. (2001). Methylation of histone H3 lysine 9 creates a binding site for HP1 proteins. Nature.

[57] Ayyanathan K., Lechner M.S., Bell P., Maul G.G., Schultz D.C., Yamada Y., Tanaka K., Torigoe K., Rauscher F.J. (2003). Regulated recruitment of HP1 to a euchromatic gene induces mitotically heritable, epigenetic gene silencing: A mammalian cell culture model of gene variegation. Genes Dev..

[58] Minc E., Courvalin J.C., Buendia B. (2000). HP1gamma associates with euchromatin and heterochromatin in mammalian nuclei and chromosomes. Cytogenet. Cell Genet..

[59] Sridharan R., Gonzales-Cope M., Chronis C., Bonora G., McKee R., Huang C., Patel S., Lopez D., Mishra N., Pellegrini M. (2013). Proteomic and genomic approaches reveal critical functions of H3K9 methylation and heterochromatin protein-1γ in reprogramming to pluripotency. Nat. Cell Biol..

[60] Morikawa K., Ikeda N., Hisatome I., Shirayoshi Y. (2013). Heterochromatin protein 1γ overexpression in P19 embryonal carcinoma cells elicits spontaneous differentiation into the three germ layers. Biochem. Biophys. Res. Commun..

[61] Huang C., Su T., Xue Y., Cheng C., Lay F.D., McKee R.A., Li M., Vashisht A., Wohlschlegel J., Novitch B.G. (2017). Cbx3 maintains lineage specificity during neural differentiation. Genes Dev..

[62] Caillier M., Thénot S., Tribollet V., Birot A.-M., Samarut J., Mey A. (2010). Role of the epigenetic regulator HP1γ in the control of embryonic stem cell properties. PLoS ONE.

[63] Brann D.H., Tsukahara T., Tau C., Kalloor D., Lubash R., Thamarai Kannan L., Klimpert N., Kollo M., Escamilla-Del-Arenal M., Bintu B. (2025). A spatial code governs olfactory receptor choice and aligns sensory maps in the nose and brain. BioRxiv.

[64] Shykind B.M., Rohani S.C., O’Donnell S., Nemes A., Mendelsohn M., Sun Y., Axel R., Barnea G. (2004). Gene switching and the stability of odorant receptor gene choice. Cell.

[65] Fuss S.H., Ray A. (2009). Mechanisms of odorant receptor gene choice in Drosophila and vertebrates. Mol. Cell. Neurosci..

[66] Bulger M., Bender M.A., van Doorninck J.H., Wertman B., Farrell C.M., Felsenfeld G., Groudine M., Hardison R. (2000). Comparative structural and functional analysis of the olfactory receptor genes flanking the human and mouse beta-globin gene clusters. Proc. Natl. Acad. Sci. USA.

[67] Sosinsky A., Glusman G., Lancet D. (2000). The genomic structure of human olfactory receptor genes. Genomics.

[68] Chen J.-H., Vanslembrouck B., Loconte V., Ekman A., Cortese M., Bartenschlager R., McDermott G., Larabell C.A., Le Gros M.A., Weinhardt V. (2022). A protocol for full-rotation soft X-ray tomography of single cells. STAR Protoc..

[69] Keenen M.M., Brown D., Brennan L.D., Renger R., Khoo H., Carlson C.R., Huang B., Grill S.W., Narlikar G.J., Redding S. (2021). HP1 proteins compact DNA into mechanically and positionally stable phase separated domains. eLife.

[70] Smothers J.F., Henikoff S. (2001). The hinge and chromo shadow domain impart distinct targeting of HP1-like proteins. Mol. Cell. Biol..

[71] Kumar A., Kono H. (2020). Heterochromatin protein 1 (HP1): Interactions with itself and chromatin components. Biophys. Rev..

[72] Hiragami-Hamada K., Shinmyozu K., Hamada D., Tatsu Y., Uegaki K., Fujiwara S., Nakayama J.-I. (2011). N-terminal phosphorylation of HP1{alpha} promotes its chromatin binding. Mol. Cell. Biol..

[73] Minc E., Allory Y., Worman H.J., Courvalin J.C., Buendia B. (1999). Localization and phosphorylation of HP1 proteins during the cell cycle in mammalian cells. Chromosoma.

[74] Bártová E., Pacherník J., Kozubík A., Kozubek S. (2007). Differentiation-specific association of HP1alpha and HP1beta with chromocentres is correlated with clustering of TIF1beta at these sites. Histochem. Cell Biol..

[75] Horsley D., Hutchings A., Butcher G.W., Singh P.B. (1996). M32, a murine homologue of Drosophila heterochromatin protein 1 (HP1), localises to euchromatin within interphase nuclei and is largely excluded from constitutive heterochromatin. Cytogenet. Cell Genet..

[76] Schott S., Coustham V., Simonet T., Bedet C., Palladino F. (2006). Unique and redundant functions of C. elegans HP1 proteins in post-embryonic development. Dev. Biol..

[77] Azzaz A.M., Vitalini M.W., Thomas A.S., Price J.P., Blacketer M.J., Cryderman D.E., Zirbel L.N., Woodcock C.L., Elcock A.H., Wallrath L.L. (2014). Human heterochromatin protein 1α promotes nucleosome associations that drive chromatin condensation. J. Biol. Chem..

[78] Muzzopappa F., Hummert J., Anfossi M., Tashev S.A., Herten D.-P., Erdel F. (2022). Detecting and quantifying liquid-liquid phase separation in living cells by model-free calibrated half-bleaching. Nat. Commun..

[79] Schoelz J.M., Riddle N.C. (2022). Functions of HP1 proteins in transcriptional regulation. Epigenetics Chromatin.

[80] Luijsterburg M.S., Dinant C., Lans H., Stap J., Wiernasz E., Lagerwerf S., Warmerdam D.O., Lindh M., Brink M.C., Dobrucki J.W. (2009). Heterochromatin protein 1 is recruited to various types of DNA damage. J. Cell Biol..

[81] Scott M.S., Calafell S.J., Thomas D.Y., Hallett M.T. (2005). Refining protein subcellular localization. PLoS Comput. Biol..

[82] Chow T.T., Shi X., Wei J.-H., Guan J., Stadler G., Huang B., Blackburn E.H. (2018). Local enrichment of HP1alpha at telomeres alters their structure and regulation of telomere protection. Nat. Commun..

[83] Smallwood A., Black J.C., Tanese N., Pradhan S., Carey M. (2008). HP1-mediated silencing targets Pol II coactivator complexes. Nat. Struct. Mol. Biol..

[84] Dialynas G.K., Terjung S., Brown J.P., Aucott R.L., Baron-Luhr B., Singh P.B., Georgatos S.D. (2007). Plasticity of HP1 proteins in mammalian cells. J. Cell Sci..

[85] Machida S., Takizawa Y., Ishimaru M., Sugita Y., Sekine S., Nakayama J.-I., Wolf M., Kurumizaka H. (2018). Structural basis of heterochromatin formation by human HP1. Mol. Cell.

[86] Wang C., Liu X., Gao Y., Yang L., Li C., Liu W., Chen C., Kou X., Zhao Y., Chen J. (2018). Reprogramming of H3K9me3-dependent heterochromatin during mammalian embryo development. Nat. Cell Biol..

[87] Kim H.-S., Roche B., Bhattacharjee S., Todeschini L., Chang A.-Y., Hammell C., Verdel A., Martienssen R.A. (2024). Clr4SUV39H1 ubiquitination and non-coding RNA mediate transcriptional silencing of heterochromatin via Swi6 phase separation. Nat. Commun..

[88] Dobin A., Davis C.A., Schlesinger F., Drenkow J., Zaleski C., Jha S., Batut P., Chaisson M., Gingeras T.R. (2013). STAR: Ultrafast universal RNA-seq aligner. Bioinformatics.

[89] Li H., Handsaker B., Wysoker A., Fennell T., Ruan J., Homer N., Marth G., Abecasis G., Durbin R. (2009). 1000 Genome Project Data Processing Subgroup the Sequence Alignment/Map format and SAMtools. Bioinformatics.

[90] Love M.I., Huber W., Anders S. (2014). Moderated estimation of fold change and dispersion for RNA-seq data with DESeq2. Genome Biol..

[91] Buenrostro J.D., Wu B., Chang H.Y., Greenleaf W.J. (2015). ATAC-seq: A method for assaying chromatin accessibility genome-wide. Curr. Protoc. Mol. Biol..

[92] Langmead B., Salzberg S.L. (2012). Fast gapped-read alignment with Bowtie 2. Nat. Methods.

[93] Heinz S., Benner C., Spann N., Bertolino E., Lin Y.C., Laslo P., Cheng J.X., Murre C., Singh H., Glass C.K. (2010). Simple combinations of lineage-determining transcription factors prime cis-regulatory elements required for macrophage and B cell identities. Mol. Cell.

[94] Satija R., Farrell J.A., Gennert D., Schier A.F., Regev A. (2015). Spatial reconstruction of single-cell gene expression data. Nat. Biotechnol..

[95] Ekman A., Chen J.-H., Vanslembrouck B., Loconte V., Larabell C.A., Le Gros M.A., Weinhardt V. (2023). Extending imaging volume in soft x-ray tomography. Adv. Photo. Res..

[96] Parkinson D.Y., Knoechel C., Yang C., Larabell C.A., Le Gros M.A. (2012). Automatic alignment and reconstruction of images for soft X-ray tomography. J. Struct. Biol..

[97] Erozan A., Lösel P.D., Heuveline V., Weinhardt V. (2024). Automated 3D cytoplasm segmentation in soft X-ray tomography. iScience.

[98] Lösel P.D., van de Kamp T., Jayme A., Ershov A., Faragó T., Pichler O., Tan Jerome N., Aadepu N., Bremer S., Chilingaryan S.A. (2020). Introducing Biomedisa as an open-source online platform for biomedical image segmentation. Nat. Commun..

[99] Pedregosa F., Varoquaux G., Gramfort A., Michel V., Thirion B., Grisel O., Blondel M., Müller A., Nothman J., Louppe G. (2012). Scikit-learn: Machine Learning in Python. arXiv.

[100] Le Gros M.A., Clowney E.J., Magklara A., Yen A., Markenscoff-Papadimitriou E., Colquitt B., Myllys M., Kellis M., Lomvardas S., Larabell C.A. (2016). Soft X-Ray Tomography Reveals Gradual Chromatin Compaction and Reorganization during Neurogenesis In Vivo. Cell Rep..

[101] Larabell C.A., Nugent K.A. (2010). Imaging cellular architecture with X-rays. Curr. Opin. Struct. Biol..

[102] van der Walt S., Schönberger J.L., Nunez-Iglesias J., Boulogne F., Warner J.D., Yager N., Gouillart E., Yu T. (2014). Scikit-image contributors scikit-image: Image processing in Python. PeerJ.

[103] Dougherty R., Kunzelmann K.H. (2007). Computing Local Thickness of 3D Structures with ImageJ. MAM.

[104] Cox J., Mann M. (2008). MaxQuant enables high peptide identification rates, individualized p.p.b.-range mass accuracies and proteome-wide protein quantification. Nat. Biotechnol..

