# Supplementary material for: HP1β and H3K9me3 Regulate Olfactory Receptor Choice and Transcriptional Identity
Source: Int J Mol Sci. 2026 Mar 24;27(7):2958. doi: 10.3390/ijms27072958 (PMC13074089; doi:10.3390/ijms27072958)
Supplement: Supplementary file 1 [file ijms-27-02958-s001.zip › ijms-4163641-supplementary.pdf]

Supplemental Figure S1. HP1β is recruited to the OR clusters. A) HP1α and HP1β ChIP-seq signal tracks on mature sorted OSN from micro dissected zones. OR cluster from Chromosome 2 is shown as a representative example. Values are reads per 10 million. Below the signal tracks, OR genes are depicted in different colors indicating the assigned expression zone. Results are shownin duplicates and with the use of different antibodies except for HP1β zone5. Antibodies used for NativeChIP: H3K9me3 (Ab8898, abcam antibody) and ChIP HP1α (HP1 ab109028 abcam antibody) and HP1β (D2F2, Cell Signaling antivody). B) Venn diagram highlighting the number of overlapped peaks from datasets shown in "A". HP1α and HP1β Cut & Run peaks (bedtools intersect) compared with H3K9me3 ChIP peaks (macs2 callpeak). At least two replicates are shown for each condition. D) Median Intensity across OR Genes of HP1α and HP1β data sets shown on "A".

Supplemental Figure S2. HP1α, HP1β and OR expression correlation analysis. A) RNA-seq analysis of gene expression of bulk RNA-seq in WT E17.5 embryos versus three week postnatal (PN) WT mice. Significantly changed genes are colored red (Padj < 0.05 for greater than 1.5-fold change, Wald test, n = 3). B) Box plot of bulk RNA-seq WT E17.5 embryos versus PN mice). OR-gene activation increases in postnatal mice. C) OR gene expression from bulk RNA-seq reads bioinformatically separated into zones. Log2FC WT E17.5 embryos versus PN. D) Cbx1 and Cbx5 expression analysis shows a swap in the transcriptional levels on Cbx1 vs Cbx5 during development. E) Model summarizing our correlation analysis. In embryo, Cbx5 shows higher expression levels. In postnatal mice Cbx1 vs Cbx5 expression levels swap and receptors from ventral zones are expressed at higher levels. Colors represent zones.

Supplemental Figure S3. Neither Swap Allele alone nor Heterozygous copy of Swap allele induces transcriptional changes in mOSN. RNA-Seq analysis of A) non OR gene expression or B) only OR genes, comparing sorted mOSN cells from WT vs Cbx control age-matched mice. Significantly changed genes are colored red (Padj < 0.05 for greater than 1.5-fold change, Wald test, n = 3). RNA-Seq analysis of C) non gene expression or D) only OR genes, comparing sorted mOSN cells from the Cbx control mice vs the Cbx heterozygous mice (Cbx5(f/+); Foxg1CRE). Cbx heterozygous mice contain two copies of the endogenous HP1a protein, one copy of the HP1b protein.

Significantly changed genes are colored red (Padj < 0.05 for greater than 1.5-fold change, Wald test, n = 3).

Supplemental Figure S4. No effect on OR transcriptional levels and on the number of cells co-expressing ORs in Cbx Swap mice. A) Single Cell RNA-seq from total MOE segregated by Seurat cluster IDs. Bottom, expression levels of developmental markers. B) Left, mOSNs segregated by Seurat Cluster ID. Right, mOSNs segregated by zone. C) Violin plots depicting OR scaled expressed per cell in Control and Swap single cells, faceted by developmental stage. N o differences in OR mRNA level per cell detected in the two genotypes. D) Fraction of OR counts from top OR, faceted by differentiation stage. No difference in number of cells co-expressing ORs was detected in the two genotypes.

Supplemental Figure S5. HP1β is implicated in H3K9me3 incorporation and MOE zone formation. A) Native ChIP-seq on micro dissected zone-1 vs zone-5 mature OSNs (n = 3 for each genotype), shows no changes in H3K9 methylation levels over Greek Islands. B) ATAC-seq on micro dissected zone-1 vs zone-5 showed a decrease in accessibility in repressed genes and an increase of accessibility in activated genes (n = 3 for each genotype).

Supplemental Figure S6. Zone 5 ORs shows a gradient of repression. HP1β is implicated in H3K9me3 incorporation and MOE zone formation. A) Native ChIP-seq on pure populations of sorted immature OSNs (OMP negative cells) from micro dissected zone-1 vs zone-5 tissue (n = 3 for each genotype). The Olfactory receptors genes, were bioinformatically segregated into anatomical zones. B) Native ChIP-seq on pure populations of sorted mature OSNs (OMP positive cells) from micro dissected zone-1 vs zone-5 tissue (n = 3 for each genotype). The Olfactory receptors genes were bioinformatically segregated into anatomical zones.

Supplemental Figure S7. and HP1β expression across the zones. A) RNA-Seq from micro- dissected zone1 and zone5 tissue. FACS sorted, OMP-GFP positive, mOSN cells (n = 3 mice per sample). B) Single cell RNA-Seq analysis (subset to only include mOSNs). Box plot depicting scaled expression of Cbx1 (top) and Cbx5 (bottom) from WT and Swap mice. C) UMAP analysis of Single Cell RNA-seq from whole MOE. Left, neuronal lineage colored by zone based on which OR is most highly expressed. Center and right, expression levels of Cbx1 and Cbx5, respectively.

Supplemental Figure S 8. HP1β is recruited to the OR clusters. A) HP1α (HP1 alpha ab109028 Abcam) and HP1β (D2F2, Cell Signaling) Cut and Run, on mature OSNs from Cbx control versus Swap mouse. Only tracks over OR gens receptors are shown (n = 2 for each genotype). B) Median Intensity across OR Genes for HP1α and HP1β data sets shown on "figure A". On Cbx control versus Swap mouse. Wilcoxon test was determine only for HP1α datasets, **** = p-value: 4.4816×10^{-200}. C) Venn diagram highlighting the number of overlapped peaks from Cut & Run (bedtools intersect) versus H3K9me3 ChIP peaks (macs2 callpeak).

Supplemental Figure S 9. HP1α and HP1β segregate into different compartments. A) SoRA Spinning disk high resolution microscopy images. IF on MOE sections with HP1*α* (magenta) and HP1β (green) specific antibodies. Dashed line shows localization of zoom images on iOSN and mOSN cells. Scale bar is 5µm. B) and C) IF images from maximum projections from z stacks from an B) immature foci, or C) a mature foci. HP1*α* (magenta) and HP1β (green) occupy different compartments. Z-n indicates the z stack projection number. Scale bar is 1µm. C) SoRA Spinning disk high resolution microscopy images. IF on MOE sections with an HP1*α* (magenta) and HP1β (green) specific antibodies in zone-5 Swap mouse. Dashed line shows localization of zoom images on iOSN and mOSN cells. HP1*α* does not migrate to the periphery as HP1β does in mOSNs (futher analysis shown in figure 8). Antibodies used on IF: HP1*α* (ab109028) and HP1β (ab10811). Apical and basal are used to indicate the outer and basal part of the tissue.

Supplemental Figure S10. HP1α and HP1β interaction partners. A) IF imaging on Trim28 MS candidates and staining intensity determination using FIJI and Intensity geomline.

Scale bar is 5mm. B) GSEA analysis on immuno-precipitated partners for HP1α and HP1β. FDR (False Discovery Rate). C) IF Imaging of MS candidates. Trim28 has a nuclear localization and localizes closer to the heterochromatin foci in mOSN. CDH4 has a nuclear localization and re-localizes with heterochromatin in mOSN. MacroH2A staining is reminiscent of HP1β in mOSN. Scale bar is 10mm. Antibodies used on IF: mH2A (ab208879), CDH4 (ab70469) and Trim28 (ab22553). Apical and basal are used to indicate the outer and basal part of the tissue.

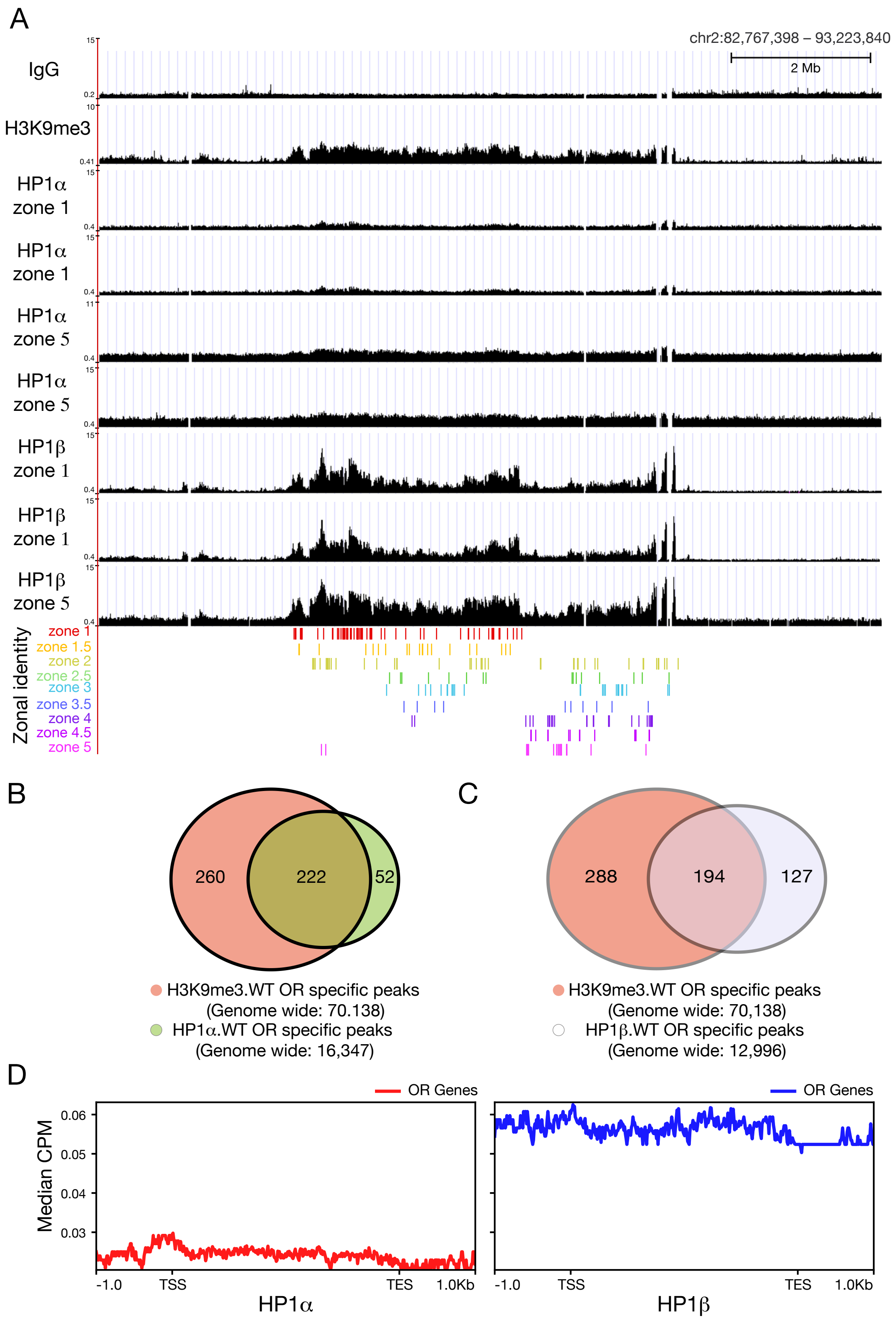
A
chr2:82,767,398 – 93,223,840
2 Mb
IgG
H3K9me3
HP1α zone 1
HP1α zone 1
HP1α zone 5
HP1α zone 5
HP1β zone 1
HP1β zone 1
HP1β zone 5
Zonal identity
zone 1
zone 1.5
zone 2
zone 2.5
zone 3
zone 3.5
zone 4
zone 4.5
zone 5
B
260
222
52
H3K9me3.WT OR specific peaks (Genome wide: 70.138)
HP1α.WT OR specific peaks (Genome wide: 16,347)
C
288
194
127
H3K9me3.WT OR specific peaks (Genome wide: 70,138)
HP1β.WT OR specific peaks (Genome wide: 12,996)
D
Median CPM
OR Genes
HP1α
HP1β
-1.0
TSS
TES
1.0Kb

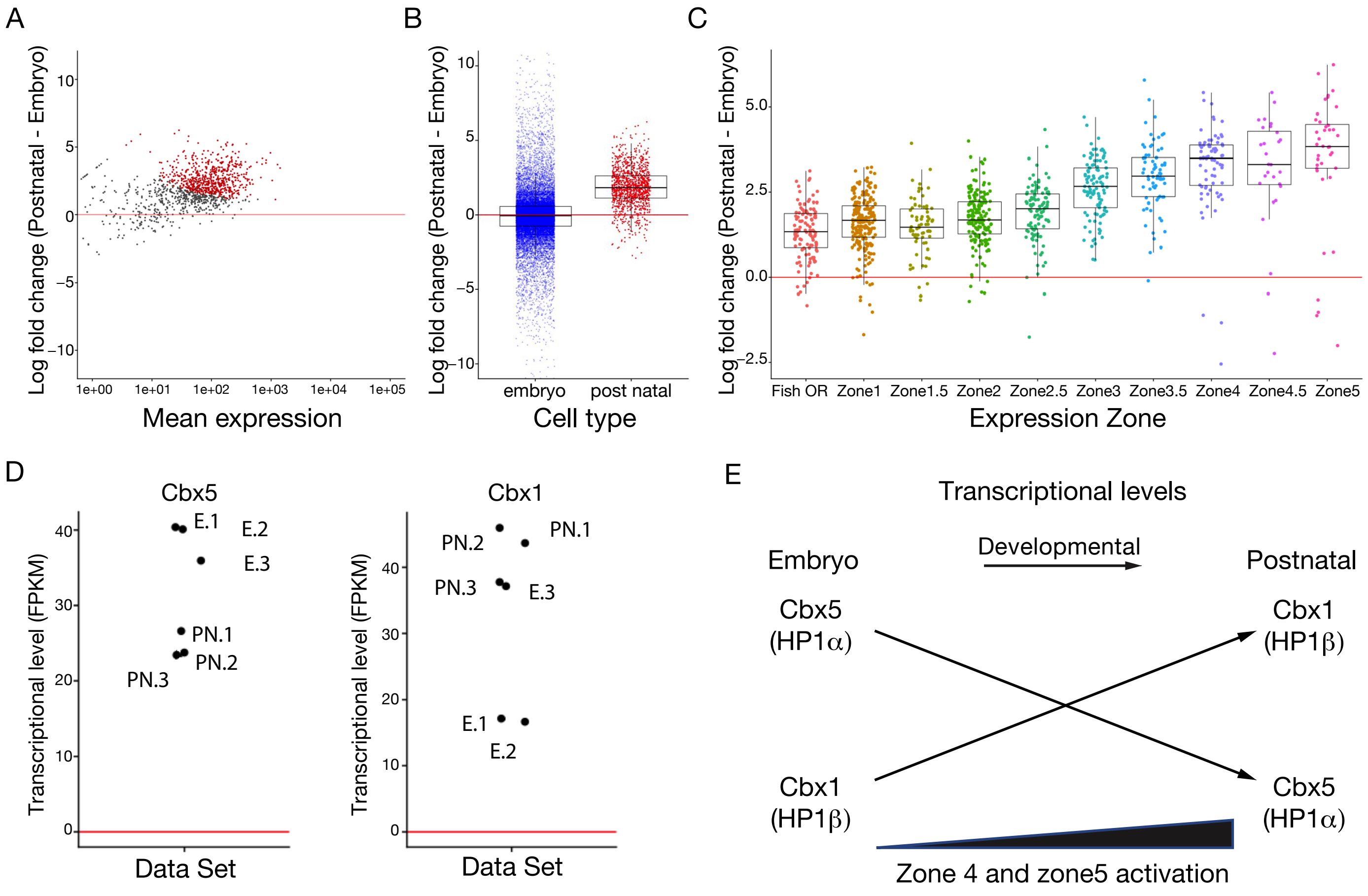
A
B
C
Log fold change (Postnatal - Embryo)
Mean expression
1e+00
1e+01
1e+02
1e+03
1e+04
1e+05
embryo
post natal
Cell type
Fish OR
Zone1
Zone1.5
Zone2
Zone2.5
Zone3
Zone3.5
Zone4
Zone4.5
Zone5
Expression Zone
D
Cbx5
Cbx1
E.1
E.2
E.3
PN.1
PN.2
PN.3
Transcriptional level (FPKM)
Data Set
E
Transcriptional levels
Embryo
Developmental
Postnatal
Cbx5
(HP1α)
Cbx1
(HP1β)
Cbx1
(HP1β)
Cbx5
(HP1α)
Zone 4 and zone5 activation

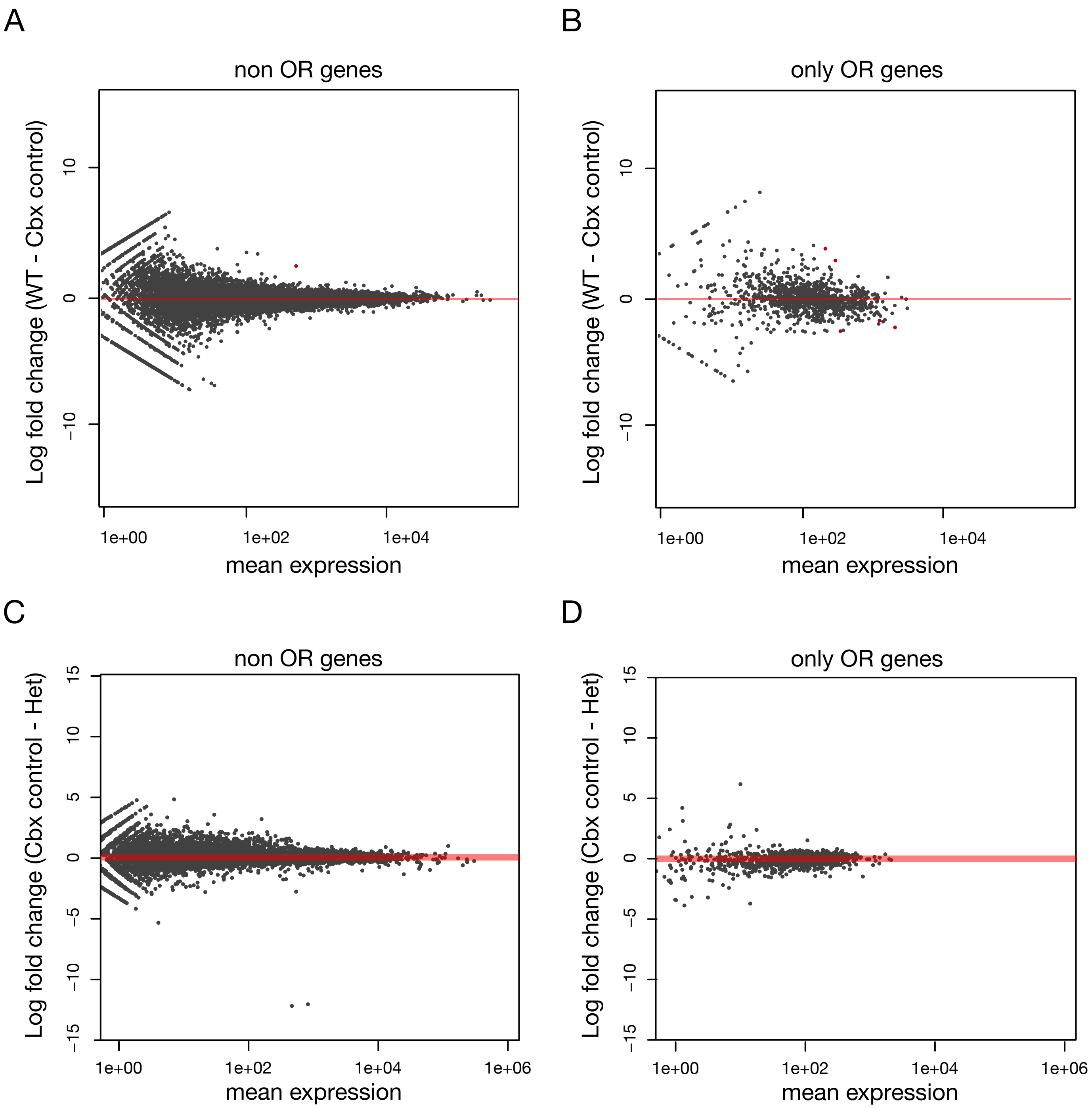
A
non OR genes
Log fold change (WT - Cbx control)
10
0
−10
1e+00
1e+02
1e+04
mean expression
B
only OR genes
Log fold change (WT - Cbx control)
10
0
−10
1e+00
1e+02
1e+04
mean expression
C
non OR genes
Log fold change (Cbx control - Het)
15
10
5
0
−5
−10
−15
1e+00
1e+02
1e+04
1e+06
mean expression
D
only OR genes
Log fold change (Cbx control - Het)
15
10
5
0
−5
−10
−15
1e+00
1e+02
1e+04
1e+06
mean expression

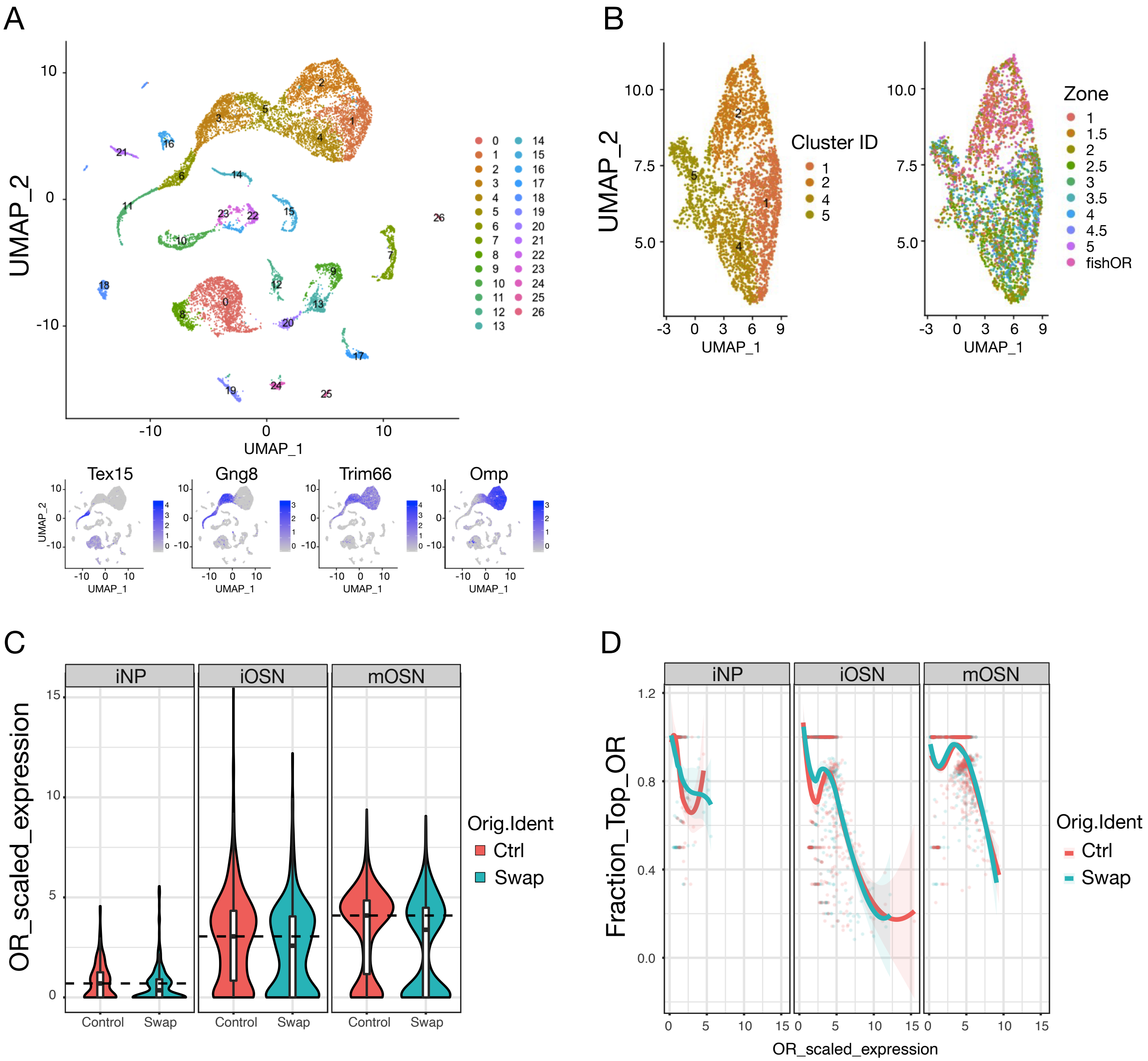
A
UMAP_2
UMAP_1
10
0
-10
0 1 2 3 4 5 6 7 8 9 10 11 12 13
14 15 16 17 18 19 20 21 22 23 24 25 26
Tex15
Gng8
Trim66
Omp
B
UMAP_2
UMAP_1
10.0
7.5
5.0
-3 0 3 6 9
Cluster ID
1
2
4
5
Zone
1
1.5
2
2.5
3
3.5
4
4.5
5
fishOR
C
OR_scaled_expression
iNP
iOSN
mOSN
Control
Swap
Orig.Ident
Ctrl
Swap
D
Fraction_Top_OR
OR_scaled_expression
1.2
0.8
0.4
0.0
0 5 10 15
iNP
iOSN
mOSN
Orig.Ident
Ctrl
Swap

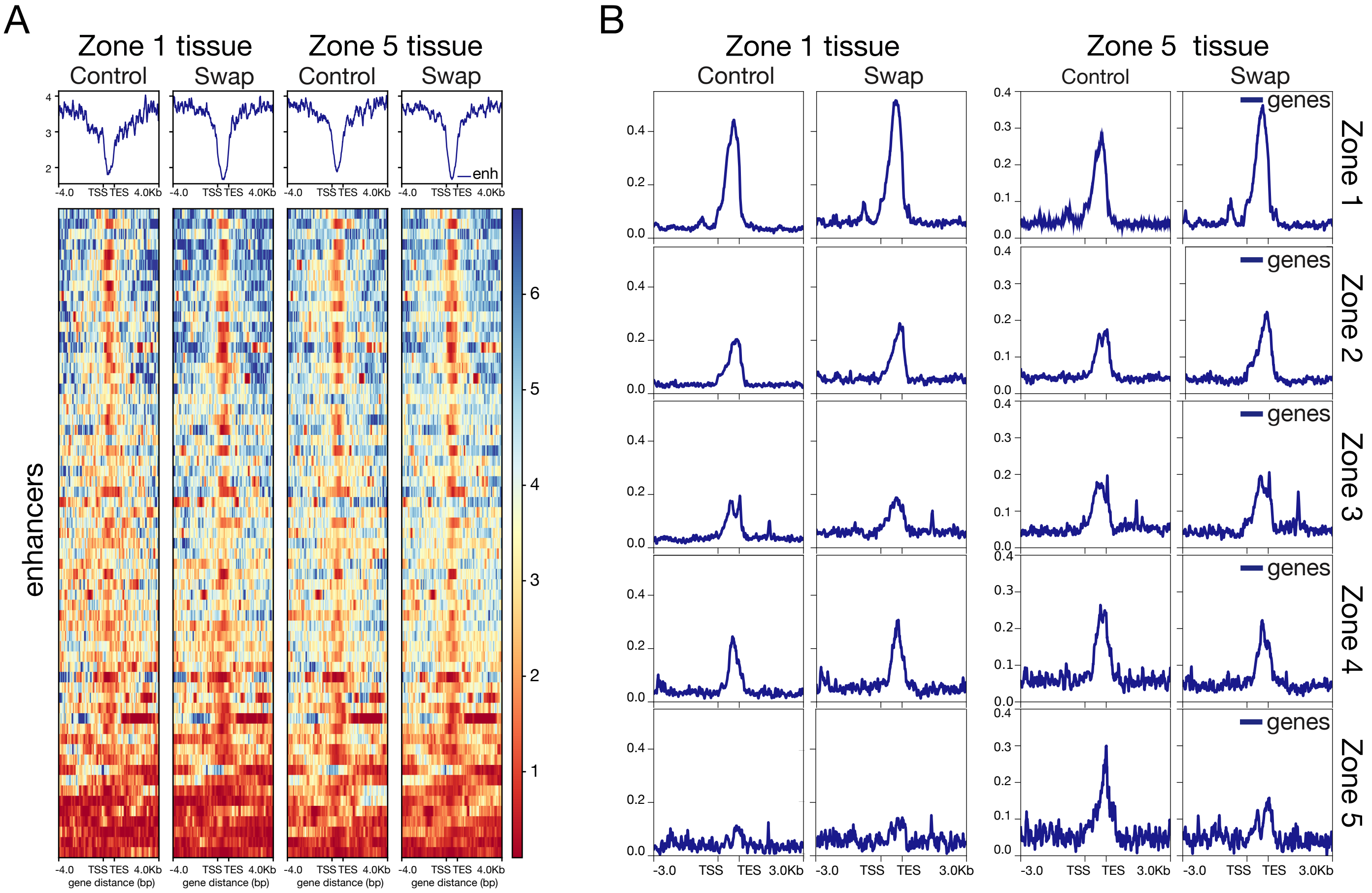
A
Zone 1 tissue
Zone 5 tissue
Control
Swap
Control
Swap
enh
enhancers
-4.0
TSS TES
4.0Kb
gene distance (bp)
B
Zone 1 tissue
Zone 5 tissue
Control
Swap
Control
Swap
genes
Zone 1
Zone 2
Zone 3
Zone 4
Zone 5
-3.0
TSS
TES
3.0Kb

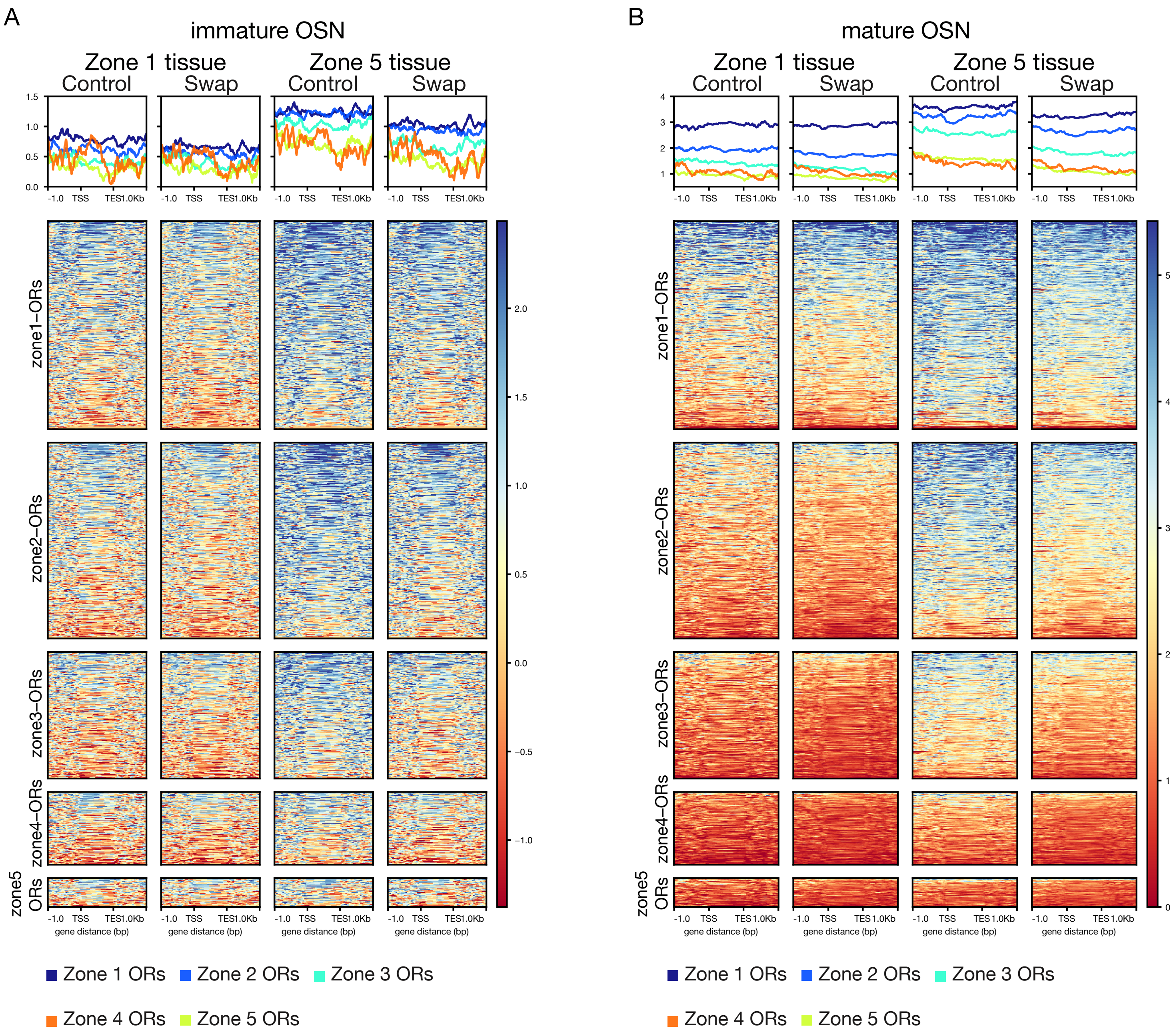

A
immature OSN
Zone 1 tissue
Zone 5 tissue
Control
Swap
Control
Swap
zone1–ORs
zone2–ORs
zone3–ORs
zone4–ORs
zone5 ORs
-1.0 TSS TES1.0Kb
gene distance (bp)
B
mature OSN
Zone 1 tissue
Zone 5 tissue
Control
Swap
Control
Swap
Zone 1 ORs
Zone 2 ORs
Zone 3 ORs
Zone 4 ORs
Zone 5 ORs

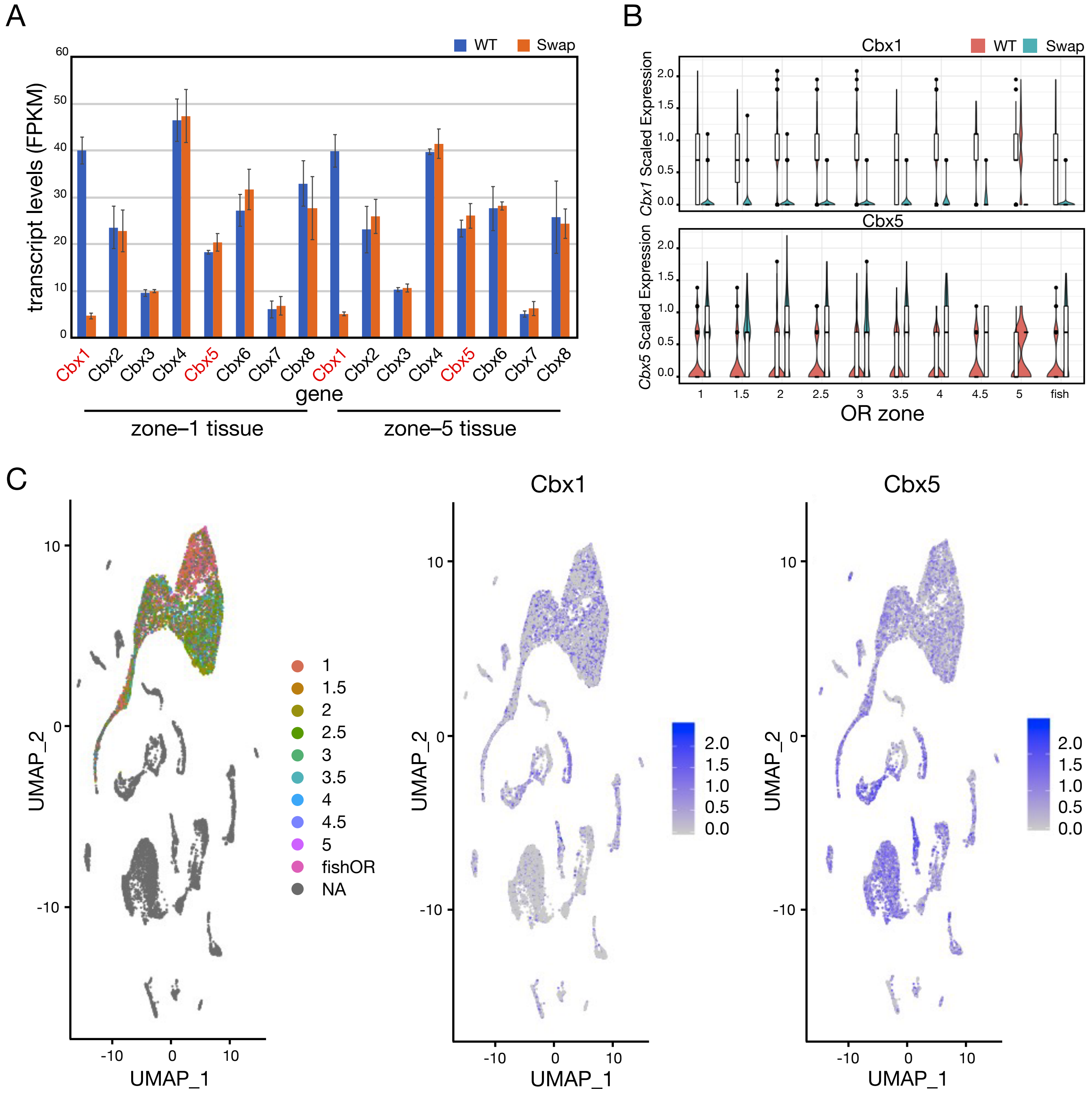

A
WT
Swap
transcript levels (FPKM)
60
50
40
30
20
10
0
Cbx1
Cbx2
Cbx3
Cbx4
Cbx5
Cbx6
Cbx7
Cbx8
gene
zone–1 tissue
zone–5 tissue
B
Cbx1
WT
Swap
Cbx1 Scaled Expression
Cbx5
Cbx5 Scaled Expression
2.0
1.5
1.0
0.5
0.0
1
1.5
2
2.5
3
3.5
4
4.5
5
fish
OR zone
C
Cbx1
Cbx5
UMAP_2
UMAP_1
1
1.5
2
2.5
3
3.5
4
4.5
5
fishOR
NA
10
0
-10

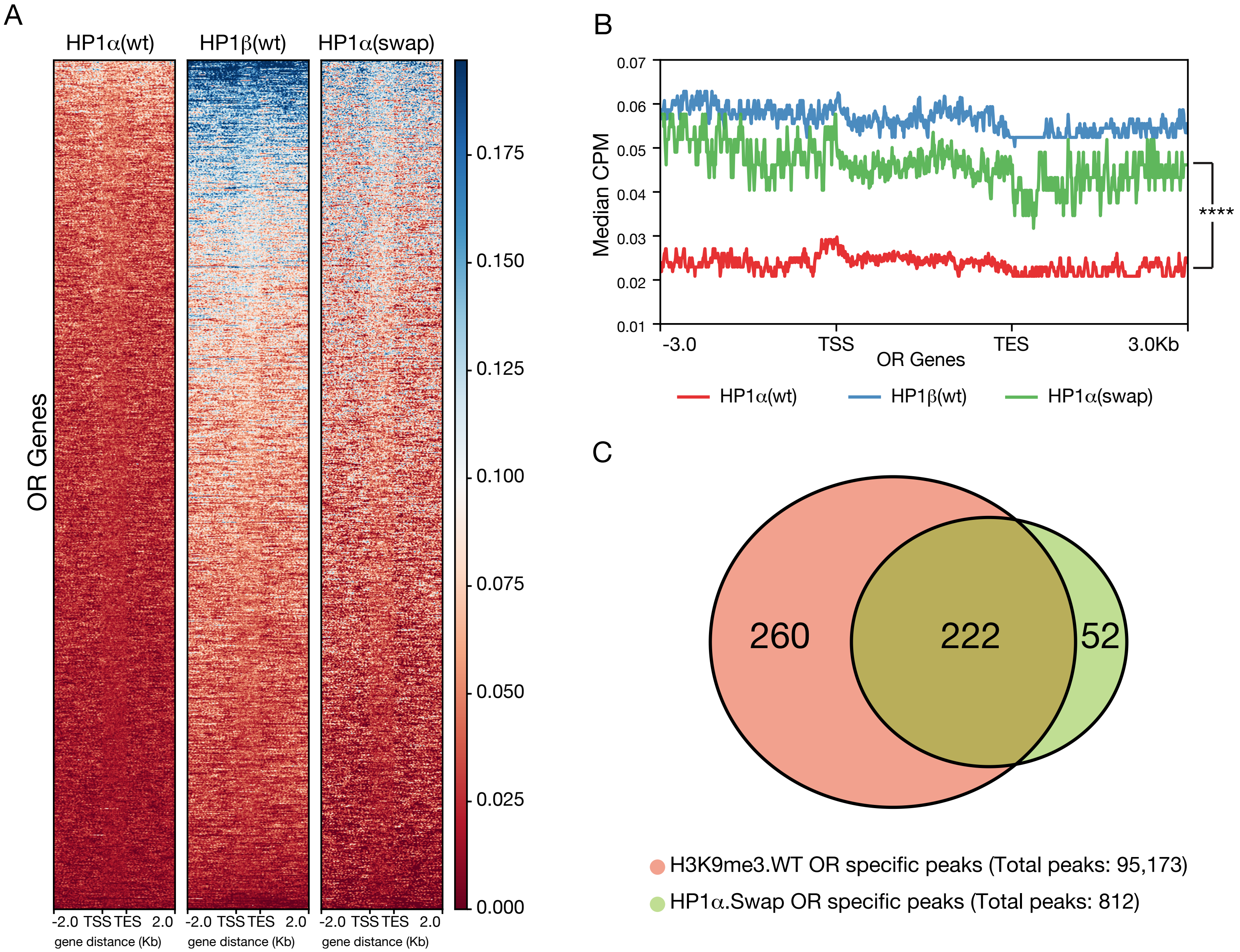
A
HP1α(wt)
HP1β(wt)
HP1α(swap)
OR Genes
0.175
0.150
0.125
0.100
0.075
0.050
0.025
0.000
-2.0 TSS TES 2.0
gene distance (Kb)
B
Median CPM
0.07
0.06
0.05
0.04
0.03
0.02
0.01
****
-3.0
TSS
TES
3.0Kb
OR Genes
HP1α(wt)
HP1β(wt)
HP1α(swap)
C
260
222
52
H3K9me3.WT OR specific peaks (Total peaks: 95,173)
HP1α.Swap OR specific peaks (Total peaks: 812)

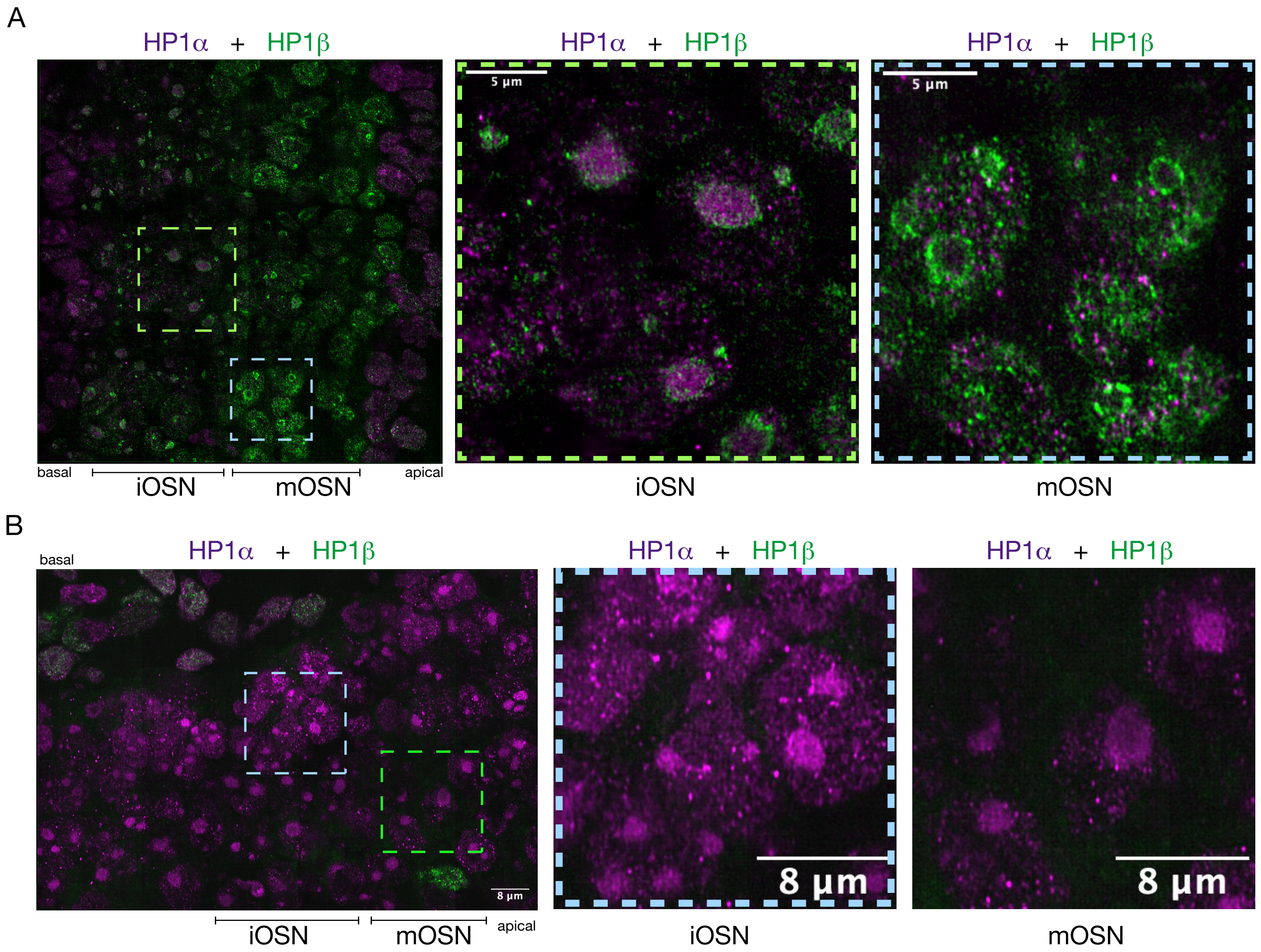

A
HP1α + HP1β
HP1α + HP1β
HP1α + HP1β
5 µm
5 µm
basal
iOSN
mOSN
apical
iOSN
mOSN
B
basal
HP1α + HP1β
HP1α + HP1β
HP1α + HP1β
8 µm
8 µm
8 µm
iOSN
mOSN
apical
iOSN
mOSN

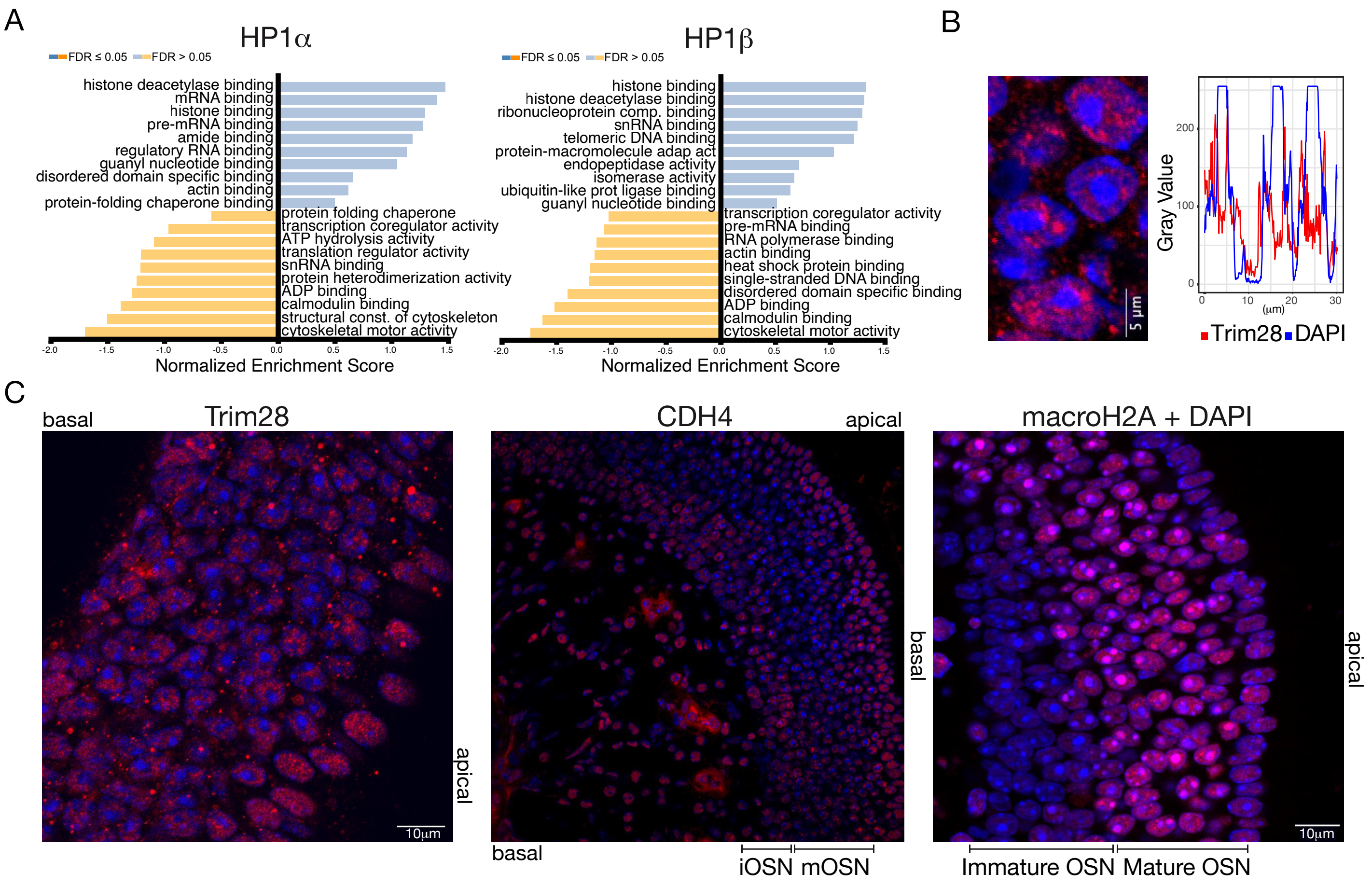
A
HP1α
FDR ≤ 0.05 FDR > 0.05
histone deacetylase binding
mRNA binding
histone binding
pre-mRNA binding
amide binding
regulatory RNA binding
guanyl nucleotide binding
disordered domain specific binding
actin binding
protein-folding chaperone binding
protein folding chaperone
transcription coregulator activity
ATP hydrolysis activity
translation regulator activity
snRNA binding
protein heterodimerization activity
ADP binding
calmodulin binding
structural const. of cytoskeleton
cytoskeletal motor activity
Normalized Enrichment Score
HP1β
histone binding
histone deacetylase binding
ribonucleoprotein comp. binding
snRNA binding
telomeric DNA binding
protein-macromolecule adap act
endopeptidase activity
isomerase activity
ubiquitin-like prot ligase binding
guanyl nucleotide binding
transcription coregulator activity
pre-mRNA binding
RNA polymerase binding
actin binding
heat shock protein binding
single-stranded DNA binding
disordered domain specific binding
ADP binding
calmodulin binding
cytoskeletal motor activity
Normalized Enrichment Score
B
5 µm
Gray Value
(µm)
Trim28 DAPI
C
basal
Trim28
apical
10µm
CDH4
apical
basal
basal
iOSN mOSN
macroH2A + DAPI
apical
10µm
Immature OSN Mature OSN
